# Silicon Photonics Transmitter with SOA and Semiconductor Mode-Locked Laser

**DOI:** 10.1038/s41598-017-14347-3

**Published:** 2017-10-24

**Authors:** Alvaro Moscoso-Mártir, Juliana Müller, Johannes Hauck, Nicolas Chimot, Rony Setter, Avner Badihi, Daniel E. Rasmussen, Alexandre Garreau, Mads Nielsen, Elmira Islamova, Sebastián Romero-García, Bin Shen, Anna Sandomirsky, Sylvie Rockman, Chao Li, Saeed Sharif Azadeh, Guo-Qiang Lo, Elad Mentovich, Florian Merget, François Lelarge, Jeremy Witzens

**Affiliations:** 1 Institute of Integrated Photonics (IPH) of RWTH Aachen University, Sommerfeldstr. 24, D-52074 Aachen, Germany; 2 III-V Lab, Campus de Polytechnique, 1 av. Augustin Fresnel, F-91767 Palaiseau Cedex, France; 3 Mellanox Technologies, Hakidma 26, Ofer Industrial Park, Yokneam, Israel; 4 Singapore Institute of Microelectronics (IME)/A*STAR, Science Park Road 11, Singapore Science Park II, Singapore, 117685 Singapore; 5 Present Address: Innolume GmbH, Konrad-Adenauer-Allee 11, Dortmund, D-44263 Germany; 6 3SP Technologies S.A.S., Route de Villejust, Nozay Cedex, F-91625 France; 7 Almae Technologies, Site Data 4, Route de Nozay, Marcoussis, F-91460 France

## Abstract

We experimentally investigate an optical link relying on silicon photonics transmitter and receiver components as well as a single section semiconductor mode-locked laser as a light source and a semiconductor optical amplifier for signal amplification. A transmitter based on a silicon photonics resonant ring modulator, an external single section mode-locked laser and an external semiconductor optical amplifier operated together with a standard receiver reliably supports 14 Gbps on-off keying signaling with a signal quality factor better than 7 for 8 consecutive comb lines, as well as 25 Gbps signaling with a signal quality factor better than 7 for one isolated comb line, both without forward error correction. Resonant ring modulators and Germanium waveguide photodetectors are further hybridly integrated with chip scale driver and receiver electronics, and their co-operability tested. These experiments will serve as the basis for assessing the feasibility of a silicon photonics wavelength division multiplexed link relying on a single section mode-locked laser as a multi-carrier light source.

## Introduction

A number of technologies are available for compact integration of photonic transceivers, prominently the Indium Phosphide (InP)^1^ and Silicon Photonics (SiP) platforms^2^, that both allow the monolithic integration of passive and electro-optic functionalities on a single Photonic Integrated Circuit (PIC). Both platforms have strengths and weaknesses: While the InP platform enables the monolithic integration of light sources (lasers, semiconductor optical amplifiers) in addition to electro-optic modulators, photodetectors and passive devices, SiP, due to its larger index contrast, enables smaller devices and may provide significant long term cost-reduction as volumes scale up, due to its compatibility with silicon CMOS manufacturing technology. Due to the absence of losses at the laser/PIC interface (monolithically integrated laser) and its ability to regenerate light within a single chip, the InP platform has enabled the chip scale integration of very complex coherent Wavelength Division Multiplexed (WDM) systems^3,4^. The SiP platform has found applicability with low cost, short distance Datacom transceivers^5^, as well as high grade coherent receivers in which the implementation of complex passive PICs (polarization diverse 90° hybrid) in a small form factor enabled by SiP device miniaturization has proven very attractive^6^. The implementation of WDM systems poses particular challenges in both InP and SiP technologies, in that frequency-selective devices are very temperature sensitive due to the high thermo-optic coefficients of semiconductors (as opposed to Planar Lightwave Circuits (PLCs) relying on dielectric materials). Moreover, fabrication variations further result in phase errors that need to be actively compensated inside WDM multiplexers in the absence of suitable trimming mechanisms. These are further exacerbated in the Silicon Photonics platform due to the high index contrast increasing the effect of waveguide sidewall roughness. With these constraints, the implementation of WDM systems in Silicon^7–10^ becomes particularly challenging, all the more when Dense (D)WDM systems with small channel spacing are targeted.

One difficulty associated to SiP is the integration of the light source: Hybrid integration of fully fabricated III-V lasers typically relies on the assembly of a micro-package containing the laser, a ball lens and an isolator that is subsequently aligned and attached to the SiP chip^11^. Other hybrid integration approaches rely on laser flip-chipping^12,13^. The latter is not only more challenging in terms of required alignment tolerances, but also requires other approaches for isolator integration^14^. Finally, hybrid integration of III-V lasers with SiP has also been realized with so-called photonic wire-bonds^15^, which has recently led to the demonstration of an aggregate data rate of 448 Gbit/s with a single laser bar and a single SiP chip^16^. Another path for laser integration has been followed in the form of heterogeneous integration of III-V materials onto Silicon chips^17,18^. Recent progress has for example been made in the form of transfer printing of the III-V material onto Silicon^19^, allowing the use of smaller III-V chips and thus enabling cost reduction. While this approach has a number of advantages such as a potentially reduced amount of required III-V material, alignment between the laser and the SiP chip with the precision afforded by lithographic overlay (if the laser is processed after III-V attachment), hermetic sealing with the Back-End-Of-Line (BEOL) of the CMOS process, and chip-scale scalability enabled by tiny lasers, it also poses a number of challenges: Introduction of III-V materials into a CMOS fabrication facility poses contamination issues, heat sinking of the lasers through the SiP chip is problematic, in particular due to the high thermal resistance of the buried oxide (BOX). This problem is exacerbated when a large number of lasers are heterogeneously integrated on the same chip, as in the case of a WDM system, thus introducing limitations to scalability. Furthermore, yield issues might be exacerbated since non-yielding lasers will result in the entire SiP chip, now combined with a number of III-V lasers, to be thrown away, while in the case of hybrid integration a known good laser die and a known good SiP die are assembled to each other. Approaches to directly grow lasers onto SiP chips either with III-V^20^ or with group IV materials^21,22^ are still in their infancy.

In the context of hybrid laser integration into SiP WDM systems, a number of options are available for the choice of an adequate light source. One possibility is to use an array of Distributed Feedback (DFB) lasers, each producing one of several optical carriers with high power and low Relative Intensity Noise (RIN), which are subsequently multiplexed into a single bus waveguide^23^. However, in this case each carrier needs to be independently controlled in order to maintain its spectral alignment to a fixed grid. Moreover, an on-chip multiplexer can result in significant additional Insertion Losses (IL) and fitting a large number of lasers into a single module might result in high cost. Another solution consists in using a semiconductor Mode-Locked Laser (MLL) producing a frequency comb with a fixed carrier spacing^24,25^. Since all the wavelengths are already multiplexed in a single beam at the output of the MLL, the use of frequency selective modulators appears a natural choice for implementing a WDM transceiver without the need of WDM (de-)multiplexers. Such a modulator is given by Resonant Ring Modulators (RRMs). Since the first demonstration of an SiP RRM in 2005^26^, a thorough understanding of the device’s characteristics has been achieved^27,28^ enabling application specific optimization and reconfiguration^29^. Utilization of RRMs in high-speed WDM interconnects in particular^30^ appears a natural application leveraging their wavelength selectivity, compactness and high speed.

The combination of an MLL with an RRM stands out by its compactness and conceptual simplicity, however the limited line power and increased RIN associated with MLLs has to be accommodated in the link budget. The line power is of particular importance in SiP, since Silicon (Si) does not have a direct bandgap and, consequently, the light has to be coupled from a hybridly^11–13^ or heterogeneously^17–19^ integrated III-V semiconductor laser. Hybrid integration in particular, while enabling the integration of a state-of-the-art known good laser die, also results in additional optical interfaces and associated power losses. While significant progress has been made in the design of more efficient optical couplers^31–34^, hybrid integration is still associated with significant optical losses compounded by further losses incurred in downstream SiP fiber-to-chip couplers. Therefore, when selecting a hybridly integrated MLL as a light source with relatively low power per carrier, downstream amplification can become a necessity. Since Erbium Doped Fiber Amplifiers (EDFA) are bulky, power hungry and expensive devices, Semiconductor Optical Amplifiers (SOA) are considered here as a possible solution offering sufficient gain and output power. However, in multi-channel operation, nonlinear effects occurring in SOAs have then also to be taken into account in addition to excess noise resulting from Amplified Spontaneous Emission (ASE).

It should be noted here that other types of comb sources, such as comb sources generated by nonlinear effects in integrated resonators^35,36^, have similar advantages in that the generated carriers are on a perfect grid, while also providing low noise comb lines when operated in the right regime^37,38^. This has enabled extremely high spectral efficiencies and aggregate data rates. However, to date, such comb sources still require pump lasers with substantial power levels (when integrated and waveguide coupled) as well as complex stabilization schemes. The primary target of the work presented in this paper consists in the implementation of DWDM functionality in short distance intra-datacenter links for which cost and power efficiency are primary metrics, so that a semiconductor single section passively mode-locked MLL^39^ appeared the better choice of light source. Also, in order to maintain low latency at short intra-data-center distances, this work is aimed at avoiding the need of Forward Error Correction (FEC) as much as possible.

While most of the work reported in this paper focuses on the Transmitter (Tx) and on the interplay between MLL, RRM and SOA in an SiP based solution, we also investigate a full link with a Receiver (Rx) also implemented in SiP in the same technology line. While the SiP Rx used for this experiment does not yet incorporate polarization management or demultiplexing functionality, it provides a good vehicle to characterize and extrapolate the link budget. On the Rx side both Germanium (Ge) photodiodes^40^ and commercial grating coupled Flip-Chip Photodiodes^41^ (FC-PD) have been used. Integrated Ge photodiodes have some advantages, such as i) having one fewer optical interface (in a multi-λ Rx with integrated SiP demultiplexer) resulting in lower IL, as well as ii) having a lower capacitance due to their small size and reduced parasitics resulting from monolithic integration. The reduced capacitance can be leveraged to increase the sensitivity of a co-designed Transimpedance Amplifier (TIA)^42^. However, Ge photodiode responsivity rapidly drops at wavelengths above ~1560 nm as determined by the direct bandgap of Ge. While the latter is narrowed by tensile strain resulting from the thermal coefficient mismatch with Si during post growth cooling of the material^43^, thus extending the cutoff of the photodiode towards longer wavelengths, this cutoff remains close to the wavelength range of interest for the utilized MLL technology. On the other hand, FC-PD based on lower bandgap III-V materials can cover a wider wavelength range, for example 1260 to 1620 nm for typical InGaAs/InP photodiodes^44^. Both receivers with integrated Ge Waveguide Photodetector (WPD)^45^ and with off-the-shelf FC-PD are characterized here.

A number of options exist for integration with electronics, spanning wire bonding^9,40,41^, flip-chip integration^10^ and monolithic integration^5^. While monolithic integration and micro-bump bonding result in reduced parasitics and facilitate architectures such as distributed drivers^46^, they are also less advantageous from the perspective of thermal management since all the heat sources are combined on a single chip increasing the thermal exposure of the laser. In this paper, 25 Gbps compatible modulator driver and TIA chips from Mellanox Technologies specifically developed for Datacom applications and verifying Quad Small Form Factor Pluggable (QSFP) electrical signaling specifications are hybridly integrated via wire bonding. Importantly, due to limitations of the current hardware, only single channel operation was experimentally verified, wherein the comb line utilized as an optical carrier was sequentially swapped in order to characterize the performance with other comb lines. These experiments serve as the basis of a calibrated link budget reported in ref.^47^, in which the penalties associated to multi-channel operation, in particular as associated to SOA nonlinearities and channel cross-talk, are reintroduced.

The paper is divided into three parts: The first part focuses on the Tx, its characterization with a semiconductor MLL as a light source as well as interoperability with wire bonded driver electronics. The second part of the paper treats the Rx integration and noise floor measurements. In the third part, a full link (SiP Tx + SOA + SiP Rx) is experimentally characterized in single-channel configuration and a comprehensive single-channel link model verified against experiments. Details of the utilized optical and Electro-Optical (E/O) components (MLL, RRM and Ge WPD) can be found in the Supplementary Materials.

## Transmitter

### Characterization with MLL Light Source and Test Equipment Grade Electronics

**Figure 1:**
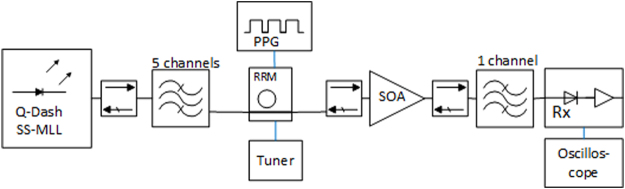
Test setup including the MLL, RRM chip and commercial SOA.

Figure 1 shows the test setup used to characterize a Tx chip using an MLL with a 102.6 GHz Free Spectral Range (FSR) as light source. After coupling the MLL to a lensed fiber and sending the light through an isolator, five comb lines are selected by a commercial wideband filter with a tunable center frequency (with less than 0.5 dB excess attenuation across its passband in addition to the 1.4 dB baseline IL of the filter). The light is subsequently grating coupled to a bus waveguide on an SiP chip comprising a single RRM (described in details as RRM chip 1 in the Supplementary Materials). The PRBS signal is generated by a Programmable Pattern Generator (PPG) and applied to the ring using microwave probes. After outcoupling from the SiP chip with a second Grating Coupler (GC), the light is sent to a commercial single polarization quantum well SOA (Thorlabs S9FC1004P) interleaved between two additional isolators. The SOA connected to the two up- and downstream isolators and operated with a 650 mA drive current at 25 °C is measured as having 24 dB gain, 15 dBm output saturation power at the −3 dB gain compression point, and a Noise Figure (NF) of 10 dBe. At 650 mA current, the bare SOA die is specified by Thorlabs as having 30 dB gain, a NF of 6 dBe and a saturation power of 18 dBm, consistent with 3 dB chip-to-fiber coupling losses at both ports, including the ILs of the isolators. As a standard Rx, a commercial 40 GHz optical passband filter with a tunable center frequency (2.4 dB IL) is used to isolate a single modulated line that is then sent to a commercial 40 GHz bandwidth photoreceiver (Finisar/U2T XPRV2021A) with an input referred TIA noise density specified to be below $40pA/\sqrt{Hz}$ and a photodiode DC responsivity specified to be between 0.5 and 0.75 A/W. The RRM was driven in reverse bias with a 2 V_pp_ drive signal.

The resonant frequency of the RRM was adjusted with a thermal tuner to sequentially modulate 4 comb lines, after which the center frequency of the wideband filter was shifted by 3.2 nm in order to test the next group of 4 comb lines. For each modulated line, eye diagrams were visualized on a real-time oscilloscope with the analog bandwidth set to 11 and 21 GHz, respectively for 14 Gbps and 25 Gbps measurements. The signal Q-factor (*Q*_*sig*_), the standard deviations (std. devs.) of the 0- and 1-level noise, the eye height and the Inter-Symbol Interference (ISI) penalty were extracted by post processing the complete signal traces. Since a large number of complete PRBS 2^7^–1 cycles were recorded (respectively 120 and 213 cycles at 14 and 25 Gbps), averaging over multiple cycles allowed separating ISI from noise during the analysis. This resulted, at 25 Gbps, in measured ISI penalties between 0.9 and 1 dB (partially accounted for by the Tx, but also partially due to the 21 GHz bandwidth limitation of the oscilloscope used to image the eye diagrams from which the ISI penalty was extracted), while at 14 Gbps ISI penalties were negligible.

Optical carrier detunings, i.e., the frequency difference between the optical carrier and the resonance of the ring, were independently optimized for 14 and 25 Gbps operation in order to obtain the best possible *Q*_*sig*_ and resulted in measured extinctions of respectively 11 dB and 6.5 dB, as well as measured on-chip modulation penalties (MPs, defined here as 10 *log*_10_((*P*_1_ − *P*_0_)/*P*_*in*_), wherein *P*_1_/*P*_0_ are respectively the power inside the bus waveguide right after the modulator when switched into the 1- and 0-states, and *P*_*in*_ is the power in the bus waveguide right before the modulator) of respectively 8.4 dB (*P*_1_ attenuated by 8 dB) and 6.9 dB (*P*_1_ attenuated by 5.8 dB). The corresponding optical carrier detunings are estimated as 5.1 GHz and 8.1 GHz; the corresponding RRM E/O cutoff frequencies (at which the E/O S_21_ drops by 3 dBe) as 18 GHz and 22 GHz. The shifting of the optimum operating point towards a higher detuning (with lower extinction) at 25 Gbps is due to the need for more bandwidth^27^ (see Fig. SM5 in the Supplementary Materials). The cumulative GC ILs and waveguide losses amounted to 10.3 dB (where it should be noted that GC ILs have since been improved to 3.5 dB per grating coupler in later chips fabricated in the same process, and further reduced to 3 dB after permanent attachment with an index matched epoxy).

It should be noted that since the experiment was done with a chip containing only one RRM, only the modulated comb line was attenuated by the RRM (corresponding to an additional 10.7 and 7.9 dB average power attenuation at respectively 14 and 25 Gbps), resulting in a large amount of excess power being sent through the SOA. This actually resulted in an average SOA input power on the order of −5 dBm, slightly above the *input* saturation power at 3 dB gain compression (−6 dBm). Unfortunately, this is not sufficient to experimentally investigate the effect of SOA saturation on channel count scalability in multi-channel operation, as important SOA saturation related penalties are highly dependent on the other channels also being modulated^47^. While the configuration used in this experiment accounts for reduced average SOA gain, increased NF^48^, as well as possible effects of joint SOA amplification on the RIN of individual comb lines^24^ (no significant increase of RIN was observed other than expected from ASE^49^), it does not account for the dynamic variations of SOA gain resulting from data being applied to the other channels (i.e., cross-grain modulation). Multi-channel operation is modelled in ref.^47^ based on the data obtained here.

**Figure 2:**
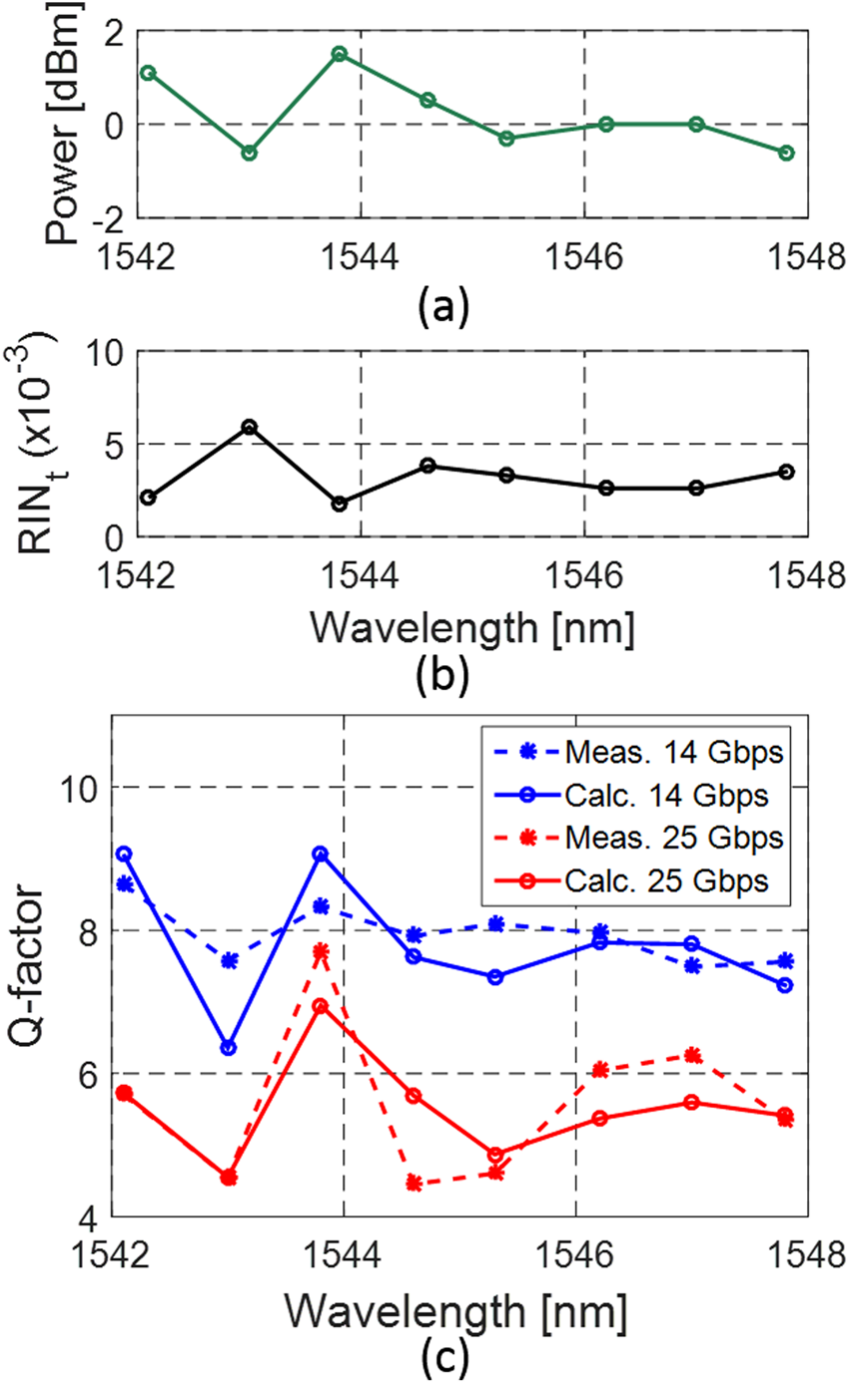
(**a**) Power and (**b**) integrated RIN for the eight consecutive MLL lines used for the Tx characterization. (**c**) Measured and calculated *Q*_*sig*_ for these eight carriers after modulation with an RRM from chip 1 with a 2 V_pp_ drive signal. The RIN shown in (**b**) was integrated between 5 MHz and 20 GHz.

For the eight modulated comb lines, Fig. 2 gives an overview of comb line power (a), aggregate single (isolated) comb line RIN, as integrated between 5 MHz and 20 GHz (b), as well as the measured *Q*_*sig*_ (c) at data rates of 14 and 25 Gbps (setup shown in Fig. 1). As can be seen, all eight carriers achieve signal Q-factors of more than 7 when modulated at 14 Gbps, the criterion required to achieve an uncorrected Bit Error Ratio (BER) below 10^-12^. However, in its current configuration, the system seems to run into its performance limit at 25 Gbps as only one carrier achieves a *Q*_*sig*_ above 7, a first indication that FEC may be required at higher data rates.

When comparing Fig. 2(a) and (b) with 2(c), a strong correlation between line power and RIN on the one hand and *Q*_*sig*_ on the other hand can be seen (as well as a strong correlation between line power and RIN). As expected, the best result was achieved for the line with highest power and lowest RIN.

In order to model the link budget, the dependency of *Q*_*sig*_ on power and noise is analytically modeled. *Q*_*sig*_ is defined as

1 $${Q}_{sig}=\frac{\eta ({P}_{Rx,1}-{P}_{Rx,0})}{{\sigma }_{1}-{\sigma }_{0}}$$

where *P*_*Rx*,1_ and *P*_*Rx*,0_ are respectively the power levels of the logical 1- and 0-states at the input port of the Rx (for long strings of repeating 1 s or 0 s), *η* is the vertical eye opening penalty due to ISI, and *σ*_1_ and *σ*_0_ are the std. devs. of the level dependent noise for the logical 1- and 0-states. The main noise sources are RIN, ASE and Rx noise, therefore the std. devs. are modeled as

2 $${\sigma }_{0/1}=\sqrt{RI{N}_{t}{P}_{Rx,0/1}^{2}+{\sigma }_{ASE,0/1}^{2}+{P}_{n,Rx}^{2}\Delta {f}_{Rx}}$$

where ${\sigma }_{{ASE},0/1}$ are the std. devs. of the ASE noise for the 1- and 0-levels, ${P}_{n,{Rx}}$ is the input referred Rx noise spectral density (in $W/\sqrt{{Hz}}$), ${\rm{\Delta }}{f}_{{Rx}}$ is the Noise Equivalent Bandwidth (NEB) of the Rx (here the oscilloscope bandwidth since its transfer function is close to being square shaped) and *RIN*_*t*_ is the aggregate RIN per line calculated as

3 $$RI{N}_{t}={\int }_{f=0}^{f={\rm{\Delta }}{f}_{Rx}}{10}^{\frac{RIN[\frac{dBc}{Hz}]}{10}}df$$

with *RIN* being the measured Power Spectral Density (PSD) expressed in dBc/Hz. Since for the utilized MLL the RIN decays to its shot noise limit above 4 GHz (it has an average of −120 dBc/Hz below, see Fig. SM3(c) in the Supplementary Materials for an exemplary RIN spectrum for one of the central comb lines), *RIN*_*t*_ is insensitive to ${\rm{\Delta }}{f}_{{Rx}}$ in the range compatible with the data rates investigated here and can be taken directly from Fig. 2(b), i.e., at both 14 and 25 Gbps the signal Nyquist frequency and thus the required Rx bandwidth are well above 4 GHz, so that the entire RIN spectrum of the MLL contributes to noise in either case (above 4 GHz, the RIN drops significantly). ${\sigma }_{{ASE},0/1}$ is derived from the SOA NF and the signal levels.

Modeling the effective *Q*_*sig*_ of the RRM + MLL + SOA Tx based on Eqs (1)–(3) taking into account the oscilloscope bandwidth (${\rm{\Delta }}{f}_{{Rx}}$) and of the 40 GHz Rx optical filter passband, we obtain the curves plotted with continuous lines in Fig. 2(c). The input referred PSD of the Rx noise was independently measured to be 45 pW∙Hz^−0.5^ (consistent with the specifications of the commercial Rx) and the RIN was set according to the data shown in Fig. 2(b).

**Figure 3:**
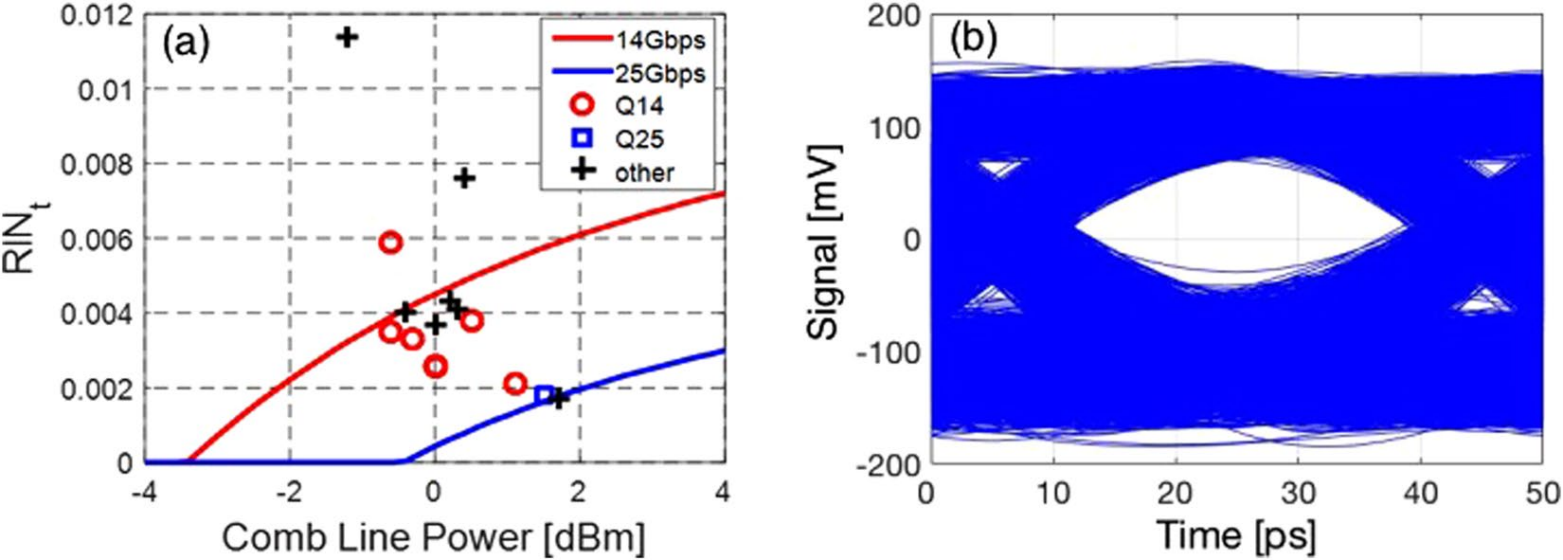
(**a**) Prediction of laser characteristics required to achieve error free (BER < 10^−12^) 14 and 25 Gbps modulation for the link architecture shown in Fig. 1 (red and blue curves) and comparison to the results of the system experiments: Markers show the experimentally recorded comb line characteristics. Red and blue markers represent the 8 consecutive comb lines with which 14 and 25 Gbps error free operation has been respectively achieved. Black markers correspond to lines for which BER has not been measured and are included to provide a complete picture of the MLL characteristics. The curves serve to classify the dots in regards to expected performance based on model predictions: dots below the red curve are predicted to reach 14 Gbps error free and dots below the blue curve are predicted to reach 25 Gbps error free. (**b**) Real time transmitter eye diagram recorded at 25 Gbps with the 1543.8 nm MLL line (blue marker in (**a**)). Note that the commercial photo-receiver used to record the eye is inverting and the optical levels have thus been flipped.

Although the resulting Q-factors follow the trend of the measurements quite well and result in the correct average Q-factor values, we see some discrepancies. These can be partially due to small variations of the RIN as a consequence of changed laser feedback resulting from slight displacements of the lensed fiber, but they are mainly attributed to a rather rough estimate of SOA input power based here on the independent characterization data shown in Fig. 2(a) rather than on an inline measurement. Overall, the model appears to be a good predictor for link budget calculations. Based on this model, we predict the maximum acceptable RIN as a function of the MLL line power for the link architecture described above (Fig. 1). The modeling results are summarized in Fig. 3 for 14 Gbps (red curve) and 25 Gbps (blue curve). The curves represent performance boundaries based on our model. Comb lines whose (power, RIN) characteristics fall below the red curve are expected to reach error free operation at 14 Gbps; comb lines whose characteristics further fall below the blue line are expected to also reach error free operation at 25 Gbps (defined in both cases as a BER below 10^−12^ without FEC).

The markers in the graph represent the characteristics of the 15 central comb lines of the MLL with power levels between −1.3 dBm and +1.7 dBm. Thus, each marker indicates the power and the RIN of an isolated comb line. The 8 consecutive comb lines characterized in the system experiments described above are colored based on whether a Q-factor of more than 7 was achieved at 25 Gbps (blue square) or only at 14 Gbps (red circles). The black crosses show the RIN and line power of the other 7 lines whose resulting *Q*_*sig*_ were not measured. They are included in the graph so that the reader may get a better idea of the overall statistics of the central comb lines’ characteristics.

Our model appears to yield a good prediction: While being a bit conservative (one line predicted to fail at 14 Gbps actually features a Q-factor above 7), the actual discrepancies between the model and the measurements remain modest (see Fig. 2(c)). Improving the cumulative GC losses by 4 dB, as already achieved in this process line, would already allow 5 lines of this MLL to reach a signal Q-factor above 7 at 25 Gbps (2 lines have almost identical power and RIN and cannot be distinguished in the graph).

### Transmitter Characterization with Hybridly Co-Integrated Driver

**Figure 4:**
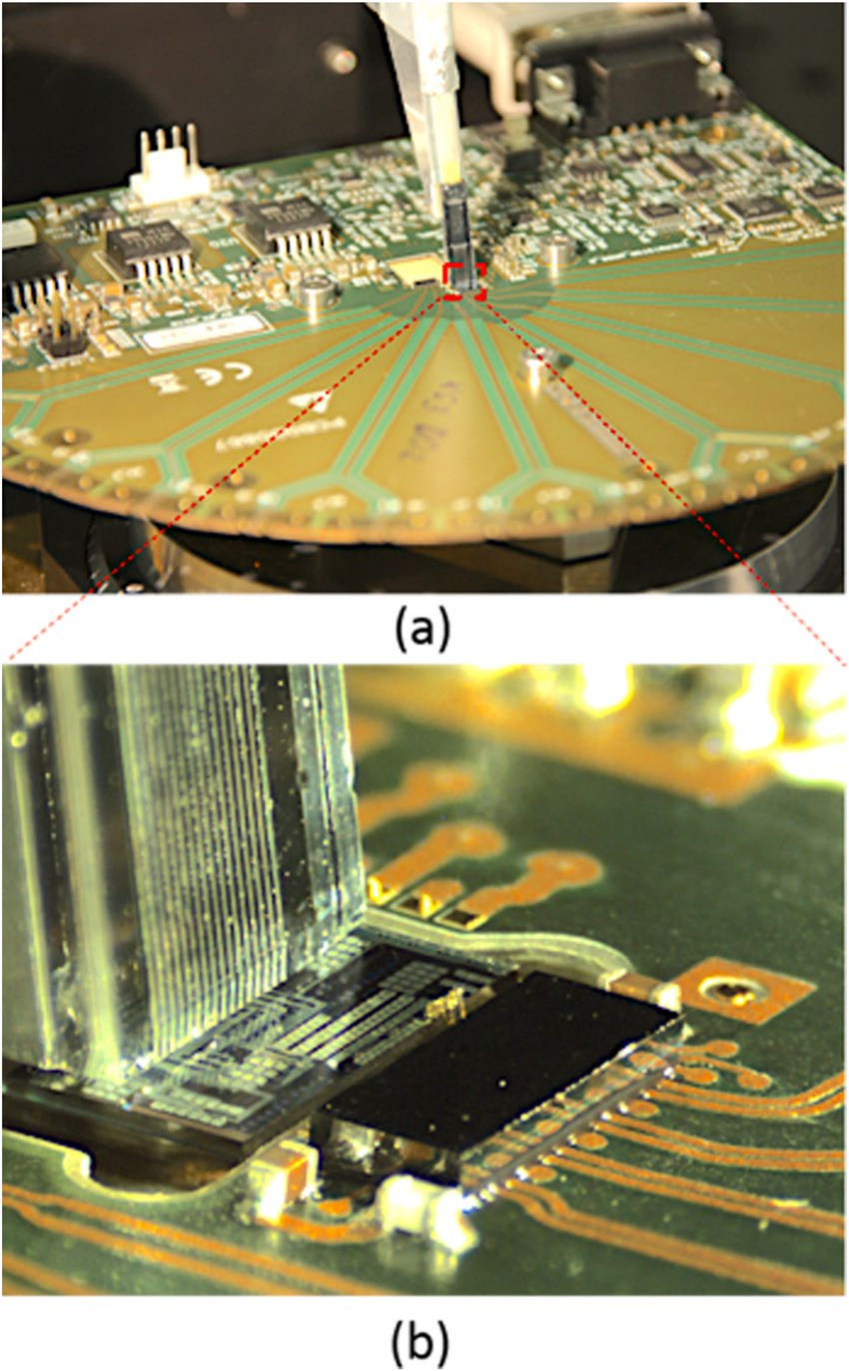
(**a**) Test board with fiber array and (**b**) detailed view of the SiP chip connected to the modulator driver with two wire bonds.

After characterizing the Tx with test equipment grade electronics connected to the SiP chip via high-speed RF probe tips, we proceed with the integration of the E/O modulator with a 25 Gbps driver chip from Mellanox technologies (part number MTxS28nn). A second Tx chip corresponding to the same design (described as chip 2 in the Supplementary Materials) was mounted on an evaluation board and a single RRM wire bonded to the driver chip (Fig. 4) with two wire bonds transporting respectively ground and signal. The SiP chip was mounted in a cavity defined in the PCB in order to ensure the height of the SiP chip to be level with the other side of the wire bonds. The distance between corresponding pads is 300 μm, the diameter of the wire bonds 25 μm and the distance between the wire bonds 100 μm. The driver supports signaling rates of up to 28 Gbd. Its output is not 50 Ω matched. Rather, it was designed to drive lumped element Ge-on-Si Franz-Keldysh direct absorption modulators with a comparable capacitance to our RRMs^50^ (the capacitance of the RRM is 39 fF at 0 V and its series resistance 53 Ω, see Supplementary Materials). It has a low output impedance of 4 Ω and consists of a self-biased differential input stage, a programmable input equalizer, a Limiting Amplifier (LA), an optional Clock Data Recovery (CDR) for high data rates, and a configurable driver output stage (set to source a 2 V_pp_ single ended signal driving the modulator between 0 and 2 V reverse bias). Pre-emphasis of the E/O channel was not needed and disabled, since the bandwidths of the E/O components are sufficient as is to sustain a 25 Gbps link.

**Figure 5:**
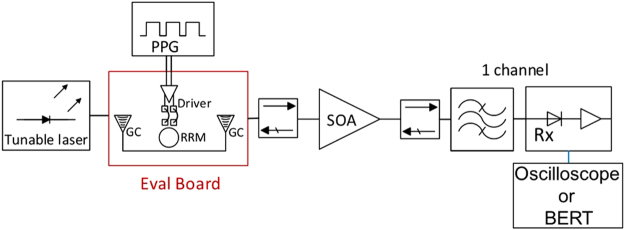
Test setup including the evaluation board with RRMs wire bonded to the modulator driver chip and commercial SOA. As previously, isolators are placed before and after the SOA.

The test setup that we used to characterize the RRM integrated with the driver chip is depicted in Fig. 5 (the 40 GHz single channel filter was replaced here by a nominally identical unit of the same model, but with reduced IL of 1.6 dB). We used a PPG from Anritsu (MU183020A 28 G/32 G bit/s PPG with 20% to 80% rise and fall times of 12 ps) to generate a PRBS 2^7^–1 data stream routed to the differential input stage of the driver. Unfortunately, the pins for accessing the thermal tuners of the RRMs were not routed off chip in this evaluation board, so that we had to use a bench-top tunable laser from Keysight Technologies (81600B) as a low noise light source instead of the MLL. Tuning of the carrier frequency was then used instead of the thermal RRM tuner to optimize the optical carrier detuning. While the higher RIN of the MLL comb lines is not taken into account in this set of experiments, interoperability between the RRMs and the driver as well as additional ISI resulting from parasitics associated with the hybrid driver integration are verified and characterized here (and found to be negligible, see below). As previously, the signal is amplified with the commercial SOA after modulation. A standard Rx is emulated with the Finisar/U2T Rx connected to either the real-time oscilloscope (with its analog bandwidth set here to 21 GHz for both 14 Gbps and 25 Gbps signal Q-factor measurements) or the 32 Gbps Error Detector (MU183040A 28 G/32Gbit/s ED) from the Anritsu Bit Error Rate Tester (BERT).

**Figure 6:**
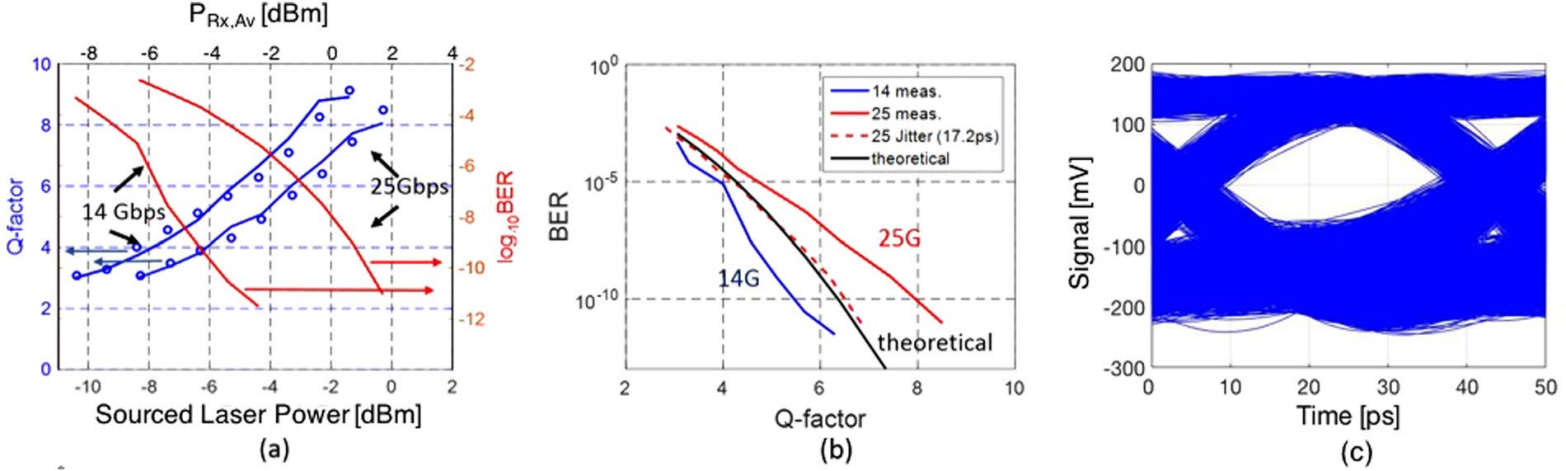
(**a**) Measured (blue points) and modeled (blue lines) signal Q-factors and measured BER (red lines) vs. sourced laser power (lower x-axis) and average power at the receiver (upper x-axis) at 14 and 25 Gbps. (**b**) Dependence of the measured BER on measured signal Q-factor for both data rates and comparison with the theoretical curve derived from Gaussian noise statistics (black line). The dashed red curve shows the modeled Q-factor at 25 Gbps assuming in addition a system jitter of 17.2 ps further discussed in the Supplementary Materials. (**c**) Real-time transmitter eye diagram recorded at 25 Gbps with 0 dBm of laser power (typical power of the isolated MLL comb lines). Note that the commercial photo-receiver used to record the eye is inverting and the optical levels have thus been flipped.

Figure 6(a) shows the measured Q-factors (blue dots) and BER (red lines) for different laser output power levels (after incurring insertion losses, the MP of the RRM and 24 dB amplification by the SOA, the average power arriving at the Rx, *P*_*Rx,Av*_, is actually ~2 dB above the power initially launched by the laser). Due to the characteristics of RRM chip 2 (lower resonator Q-factor) and the reduced rise and fall times of the chip-scale driver, the signal Q-factor turned out to be less sensitive to optical carrier detuning than in the experiments reported in the previous section in a range common to both data rates. Thus, a common optical carrier detuning of 7.5 GHz could be used for both data rates, leading to the following RRM characteristics: an extinction of 8.9 dB, an MP of 8.4 dB and a cutoff frequency of 20 GHz. ISI penalties were extracted from the recorded eye diagrams to be respectively 0.3 and 1 dB at 14 and 25 Gbps. At 14 Gbps and 25 Gbps we respectively obtained error free operation (BER < 10^−12^) at −3.4 dBm and 0.7 dBm of laser power. These data points are not indicated on the graphs as the exact BER was below the 10^−12^ floor that could be reliably measured with the chosen BERT gating period (12 min. at 14 Gbps and 7 min. at 25 Gbps, corresponding to 10X the mean time between errors at a 10^−12^ BER). Error free measurements confirm that there is no critical noise floor in the system.

Based on Eqs (1)–(3), the measured Q-factors were reconstructed as in the previous subsection. The excellent match between measured (points) and calculated (lines) Q-factors shown in Fig. 6(a) further confirms our model.

In a second step, the dependence of the BER on *Q*_*sig*_ is compared to the theoretical expectations based on the simplified assumption of Gaussian noise statistics (black line in Fig. 6(b))

4 $$BER=\frac{1}{2}erfc(\frac{{Q}_{sig}}{\sqrt{2}})$$

where *erfc* is the complementary error function. It is apparent that the measured BER is better than predicted from the Q-factors at 14 Gbps and worse than predicted at 25 Gbps. The penalty observed at 25 Gbps corresponds to 2 dBQ (defined as *Q*[dBQ] = 20*log*_10_(*Q*)) and might be partially associated to slowly varying timing jitter not recorded in the eye diagrams as discussed in the Supplementary Materials. The better than expected BER at 14 Gbps corresponding to a discrepancy of 1.2 dBQ might be partially due to the different test environments (characterization with real time oscilloscope with a square shaped transfer function vs. error detector with a larger than 30 GHz analog bandwidth introduces both a slight ISI penalty as well as a worsening of ASE-beat noise^51^ that together can account for the discrepancy). While strictly ASE noise statistics follow a chi-square distribution rather than Gaussian statistics, resulting discrepancies are expected to be very slight and not sufficient to account for observed discrepancies^52^, which we further experimentally confirmed here (see Supplementary Materials, Fig. SM10(a)).

**Figure 7:**
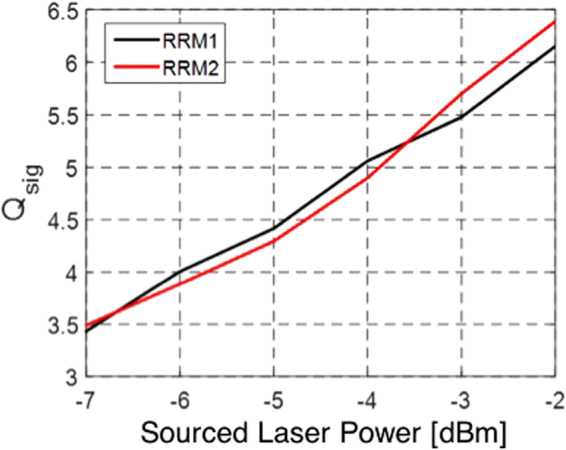
Comparison of the Q-factor measured at 25 Gbps as a function of sourced laser power for RRM1 and RRM2. RRM1 was directly probed with high-speed probe tips, while RRM2 was wire bonded to the chip-scale driver.

In order to visualize possible penalties arising from Tx integration, Fig. 7 shows a comparison of *Q*_*sig*_ at 25 Gbps as a function of sourced laser power measured with either (a) RRM chip 1 and instrument grade electronics connected to the SiP chip via probe tips or with (b) RRM chip 2 wire bonded to the driver chip. A direct comparison is made more challenging by the fact that the two modulator chips have somewhat different characteristics due to fabrication variations. While RRM1 and RRM2 were both operated with the same optical carrier detuning (7.5 GHz) for this experiment and had comparable bandwidths (the slightly higher intrinsic bandwidth of RRM2 is reduced by connectivity with the chip-scale electronics), RRM1 had both a better MP (7.1 vs. 8.4 dB, resulting in a slightly higher signal level notwithstanding slightly worse cumulative GC losses by 0.6 dB) as well as a worse extinction (7.2 vs. 8.9 dB). Even though both rings have a comparable bandwidth, the extracted 25 Gbps ISI penalty (1 dB for RRM2 vs. 0.93 dB for RRM1) is slightly higher for RRM2 pointing to a slight influence of the driver. Overall, one may conclude that the penalty associated to the driver chip integration is slight and might be associated to the somewhat higher fall and rise times of the chip-scale driver (16 ps vs. 12 ps for the PPG).

## Receiver

Two versions of the Rx were implemented and characterized: One consists of an integrated Ge WPD directly connected to a waveguide routed to a single polarization GC acting as an optical input port. The other relies on a grating coupled FC-PD, i.e., the light first enters the SiP Rx chip via a first single polarization GC, is routed to the location of the FC-PD, to which it is then routed by means of a second GC. This configuration will allow interposing a demultiplexer between the input grating coupler and the array of FC-PDs in the future, for example by means of on-chip OADMs. These were not yet implemented and were functionally replaced in the full link characterization by the off-chip, 40 GHz passband, single-channel filter also used in the previous section. Suitable OADMs developed in the same process have been shown to have on-chip insertion losses below 0.85 dB^49^, which is below the insertion losses of the external single channel filter used in section II and in the full link characterization (section IV). Co-operability of both types of photodiodes with a Rx chip from Mellanox Technologies comprising a TIA, optional channel equalization (unused here), an LA and an optional CDR (used at 25 Gbps) is verified and the sensitivity floor of the Rx measured. While polarization management was not implemented here, associated penalties are taken into account in the WDM link modelling based on these experiments^47^.

### Receiver Integration and Noise Floor Measurements

**Figure 8:**
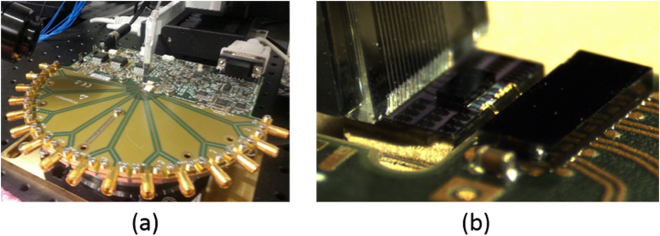
(**a**) Photograph of the Rx evaluation board. (**b**) Photograph of the SiP chip with FC-PD. The SiP chip is mounted on the evaluation board and wire bonded to the TIA + LA chip.

In order to test the interoperability of both solutions (FC-PD and Ge-PD) with the IPTA28G4CPT Rx chip from Mellanox Technologies, we mounted the SiP chips on evaluation boards and wire bonded them to the electronic Rx chip. In both cases an array of 4 photodiodes was electrically connected to the TIA + LA chip with 4 pairs of interleaved GS wires with a diameter of 25 µm bridging a distance of 200 μm between pad frames and with a distance of 130 μm between wires. As for the Tx, the Rx SiP chip was mounted in a cavity to allow the wire bond pads to be even with each other. The TIALA chip supports serial signaling rates up to 28 Gbd per channel with dynamically adjustable decision threshold (offset compensation), optional retiming (CDR), and transmission line drivers with configurable output swing and pre-emphasis to compensate for RF signal distortion in following PCB traces. Optional equalization of the E/O channel was not required and not used. The input impedance of the TIA is 50 Ω and the reverse bias applied to the photodiode 2 V. A photograph of the Rx evaluation board and a zoomed in view of an SiP + FC-PC + TIALA subassembly are shown in Fig. 8.

**Figure 9:**
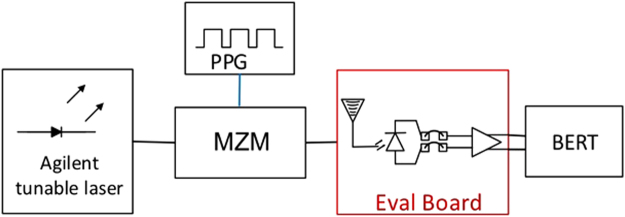
Block diagram of the measurement setup used for characterizing the Rx evaluation boards.

These evaluation boards have been characterized using the setup shown in Fig. 9. An optical carrier with a 1540 nm center wavelength is generated with the tunable laser from Keysight Technologies and is coupled into a 40 Gbps MZM from Gigoptix (LX8401) with an analog cut-off frequency of 33.5 GHz serving as a reference Tx. The data stream is generated by the Anritsu PPG (MU183020A 28 G/32 Gbps PPG) as a PRBS 2^7^–1 sequence fed into the modulator. The modulator is biased so as to achieve maximum extinction ( > 50 dB), meaning it is not exactly biased at its 3-dB operating point but at a lower average output power to accommodate the finite drive voltage. The modulated light is directly fiber coupled to the Rx SiP chip using a GC (with an improved design compared to the Tx chips and fiber coupling losses reduced to 3.5 dB without index matched epoxy). The electrical output of the evaluation board is connected to the 32 Gbps ED of the Anritsu BERT (MU183040A 28 G/32Gbit/s ED). The responsivities of the Ge WPD and of the FC-WPD are respectively 0.67 A/W and 0.84 A/W at 1550 nm, resulting in external responsivities of 0.31 A/W and 0.21 A/W as measured relative to the power in the fiber at the Rx input, due to the losses resulting from respectively 1 and 2 GCs. The cutoff frequencies of the subassemblies as measured in a 50 Ω environment were respectively in excess of 30 GHz for the Ge WPD and between 15 and 18 GHz for the FC-PD subassemblies. More details on the devices can be found in the Supplementary Materials.

**Figure 10:**
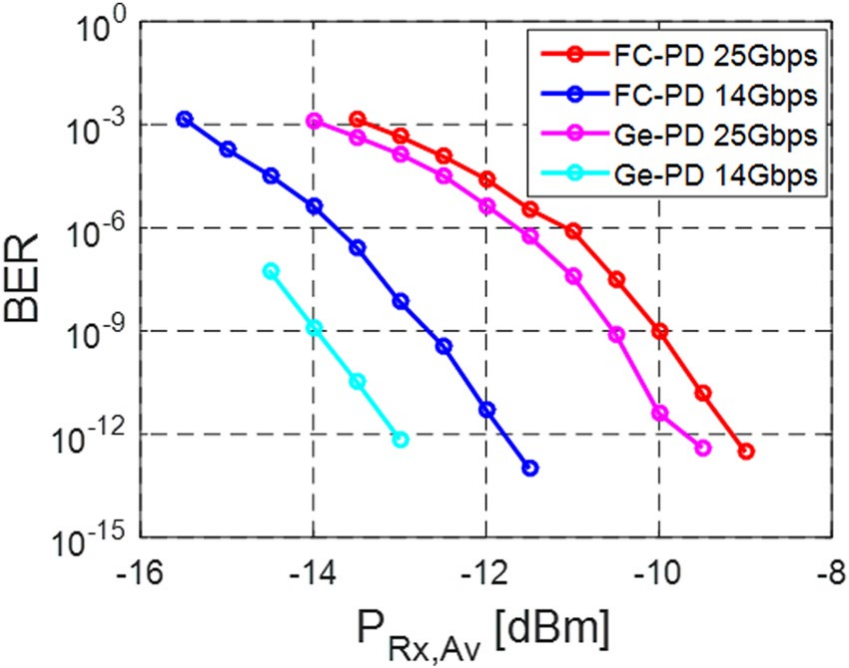
BER vs. average Rx input power at 14 and 25 Gbps for the FC-PD and Ge WPD. The reference modulator was operated with an extinction ratio above 50 dB.

Our tests (Fig. 10) show that the Ge WPD evaluation board presents better results with a sensitivity of −13 dBm at 14 Gbps (around −16.5 dBm discounting GC losses) and −9.7 dBm at 25 Gbps (−13.2 dBm discounting GC losses), defined as the average power required to achieve a BER of 10^−12^ assuming infinite extinction, i.e., ½ of the required Optical Modulation Amplitude (OMA). The FC-PC based SiP chips on the other hand feature a sensitivity limit of respectively −11.8 dBm and −9.1 dBm at 14 and 25 Gbps (average power in fiber). The performance enhancement of the Ge WPD based chip, 1.2 dB at 14 Gbps and 0.6 dB at 25 Gbps, is not quite as large as the 2 dB expected based on the external responsivities of 0.34 A/W and 0.21 A/W, respectively measured at 1540 nm for the Ge WPD and the FC-PD based Rx chips prior to wire bonding to the TIAs. This might be related to the fact that the TIA was optimized for an input load corresponding to a flip chip photodetector or to excess noise generated by the Ge WPD. It should also be pointed out that the improvement of the Ge WPD based system is not intrinsic to the integrated photodiode itself (that actually has worse performance in terms of deembedded responsivity and dark current), but rather to the fact that one less optical interface was required in a WDM compatible configuration that precludes direct coupling of the fiber to the FC-PD.

The very different sensitivities at 14 and 25 Gbps are not related to the system bandwidth, as it was the same in both cases (the bandwidth of the TIALA is not adjustable). Since the output of the TIALA chip is reshaped (and the system measurement thus less sensitive to sampling jitter in the ED) and retimed (and the system measurement thus less sensitive to upstream jitter), one would expect this penalty to be of a different nature than the one seen during 25 Gbps Tx characterization. Moreover, its magnitude is much higher here (2.65 dB, corresponding to 5.3 dBQ) so that we expect it to be primarily due to a limitation of the chip-scale Rx electronics rather than to the test environment. Differences in the magnitude of the penalty depending on whether a Ge WPD or a FC-PD is utilized is attributed to the different electrical load applied to the input of the TIALA influencing the behavior of the circuit.

### Input Referred TIA Noise and Data Rate Dependent Penalty

**Figure 11:**
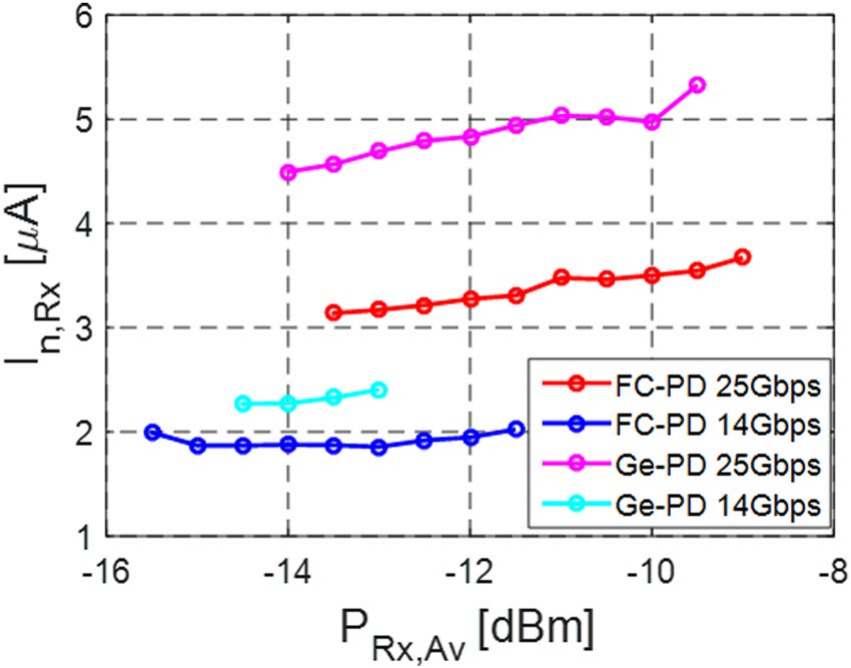
Input referred Rx noise for the FC-PD and Ge WPD photoreceivers at 14 and 25 Gbps as a function of the average Rx input power (measurement done with an extinction ratio of over 50 dB).

The BER data reported in the previous section can be used to extract an equivalent input referred Rx noise. The BER is first converted into a Q-factor according to Eq. (4), which is combined with (1) to extract the std. dev. of the Rx noise. Since the Rx noise is additive, we assume *σ*_*1*_ and *σ*_0_ to be equal. *P*_*1*_ *-P*_*0*_ is the absolute OMA (twice the average power in this case as we applied a signal with a very high extinction). We thus obtain the following expression for the std. dev. of the Rx noise

5 $${\sigma }_{Rx}=\frac{\eta ({P}_{1}-{P}_{0})}{2\sqrt{2\cdot }erf{c}^{-1}(2BER)}$$

which is multiplied by the GC insertion efficiency and the photodiode responsivity in order to obtain the input referred TIA current noise (integrated over the entire NEB of the system), *I*_*n,Rx*_. As a simplifying assumption in the extraction of the input referred noise, we assume there to be no additional ISI penalty arising inside the TIA (i.e., *η* = 1), since we cannot extract it from the reshaped signal at the output of the LA (we expect this to be an adequate assumption at 14 Gbps at which the ISI penalty should be very small). The results are depicted in Fig. 11 for both the FC-PD and the Ge WPD at 14 and 25 Gbps. The values at 14 Gbps can be taken to correspond to actual input referred noise integrated over the NEB, while the values at 25 Gbps also effectively take the penalty arising from data rate dependent signal distortion inside the Rx into account, such as e.g. bandwidth limited signal distortion or internally generated jitter (corresponding to the 2.65 dB penalty reported in the previous section).

## Full Link

After separate analysis of the Tx and Rx, this section is dedicated to the characterization and modeling of the whole link operated in back-to-back configuration, i.e., with a short polarization maintaining fiber connecting the Tx and Rx evaluation boards. The results are compared with predictions based on the Tx and Rx characteristics determined above. Due to the lack of access to the thermal tuners of the RRMs, the experimental characterization in this section also relies on the bench top tunable laser. The link penalty associated to the RIN of the MLL (as reported in section II.A) is reintroduced in the link model at the end of this section. While the primary focus of investigation was short reach transmission for intra-datacenter links, investigations of chirp induced ISI penalties for transmission distances up to 10 km can be found in the Supplementary Materials. These indicate that at the 2 V_pp_ RRM drive voltage used in the present experiments, RRM induced chirp does not play a role at the investigated 10 km fiber length (at increased 6 V_pp_ drive voltage a slight 0.5 dB vertical eye closure penalty was seen).

**Figure 12:**
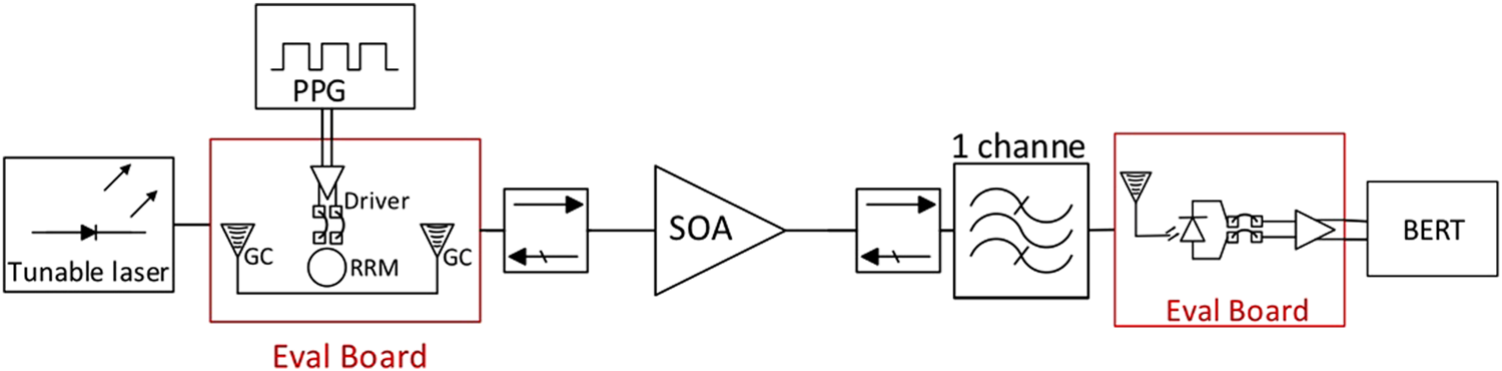
Test setup used to characterize the Tx and Rx operated together.

**Figure 13:**
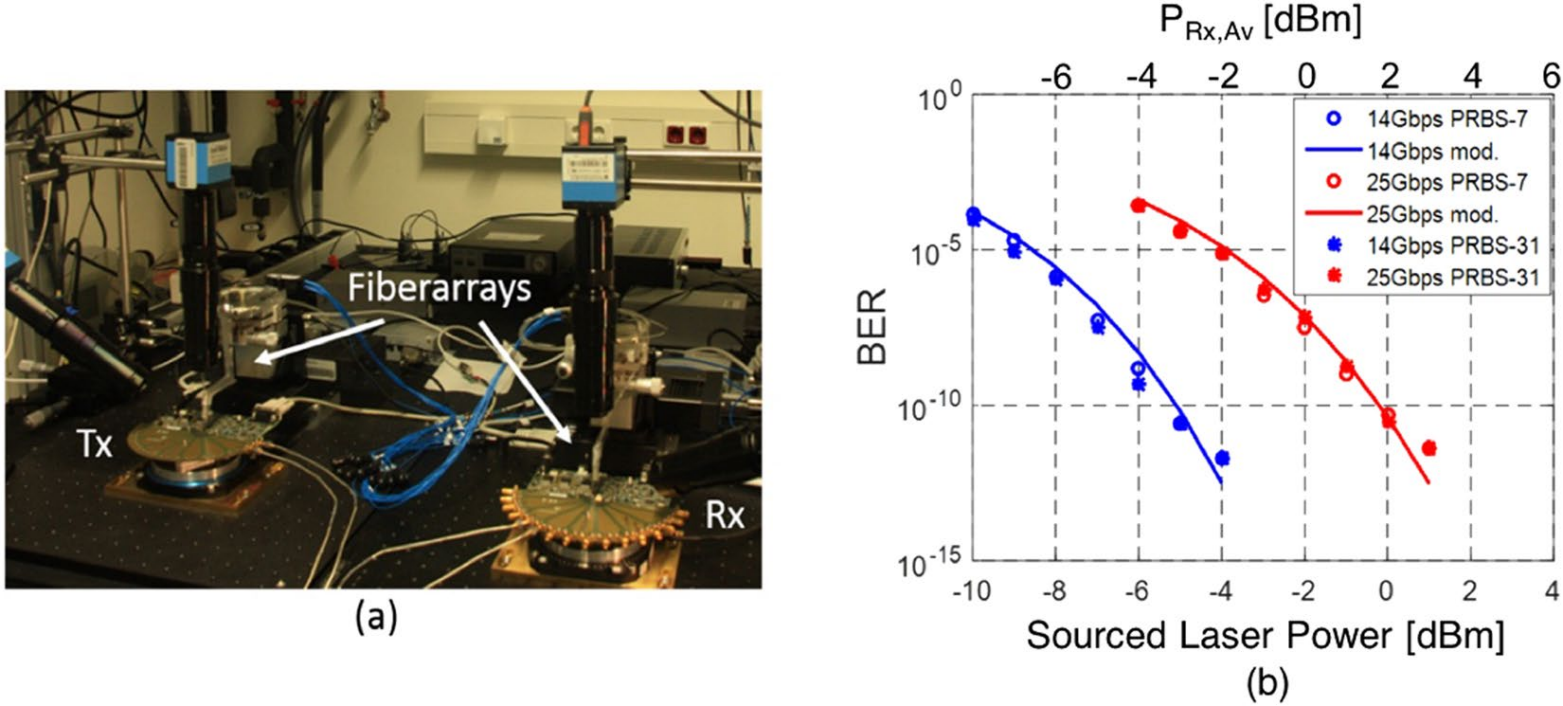
(**a**) Photograph of the test setup combining the Tx and Rx evaluation boards and (**b**) measured BER (points) as a function of sourced laser power (lower x-axis) and average power at the receiver (upper x-axis, after 24 dB amplification by the SOA), as well as predicted BER based on the link model (continuous lines). The BER is given as a function of the sourced laser power, as it is the objective of this work to assess the compatibility of the link architecture with the capabilities of semiconductor MLLs. Given the sensitivity floor of the SiP Rx (−13 dBm at 14 Gbps, −9.7 dBm at 25 Gbps), the noise budget is completely dominated by ASE and RIN, and a substantial signal attenuation could be tolerated after the SOA prior to receiver noise playing a substantial role.

Figure 12 gives an overview of the test setup used for characterizing the complete link. We are characterizing the combination of Tx, SOA and Rx with a fiber coupled tunable laser used as a light source. As a consequence, we do not need to tune the RRM. Tx chip 2 (RRM2, wire bonded to the driver chip) and the Rx version with FC-PD (wire bonded to the TIALA chip) are used here. ASE-signal beat noise is filtered by the 40 GHz optical filter and the 20 GHz NEB of the FC-PD + TIALA chip. With the setup shown in Fig. 13(a) we recorded BERs for sourced laser power levels between −10 dBm and 1 dBm at both 14 Gbps and 25 Gbps. For both data rates, we biased RRM2 at a very similar optical carrier detuning than in section II.B (8.9 dB extinction) so that the RRM characteristics reported there can also be assumed in this section.

The recorded BERs can be seen as dots in Fig. 13(b) together with a prediction (continuous line) based on the characterization results of the previous sections and modeling based on Eqs (1)–(4). In the link model, we assume the previously determined component characteristics (interface losses, RRM extinction and MP, SOA gain and NF, FC-PD responsivity) as summarized in Table 1. The input referred noise of the TIA corresponds to the data extracted at 14 Gbps from the FC-PD Rx characterization (deep blue curve in Fig. 11) and corresponds to an estimated input referred noise density of $14\,pA/\sqrt{Hz}$. An additional ISI penalty of 2.65 dB is assumed at 25 Gbps, which corresponds to the excess penalty seen at 25 Gbps in the sensitivity floor of the FC-PD Rx board (red 25 Gbps curve in Fig. 10 relative to the deep blue 14 Gbps curve). Based on these numbers, the Q-factors are calculated first and subsequently converted into a BER assuming a Gaussian noise model (Eq. (4)).

**Table 1:** List of Device and System Characteristics (Full Link).

Quantity	Value	Comment
GC IL (Tx)	4.8 dB	Per GC
RRM Ext.	8.9 dB	
RRM MP	8.4 dB	As defined in II.A.2
SOA Gain	24 dB	SOA + up & downstream isolators
SOA NF	10 dBe	SOA + up & downstream isolators
40 GHz Filter IL	1.6 dB	
FC-PD Ext. Resp.	0.21 A/W	Includes 6 dB excess Rx GC losses
TIA Inp. Ref. Noise	2 μA	Integrated over Rx bandwidth
ISI Penalty (25 G)	2.65 dB	Rx signal distortion @ 25 Gbps

As can be seen in Fig. 13(b), the model reproduces the experimental data very well. Moreover, this data shows that no additional penalty arises from jointly operating the SiP Tx with the SiP Rx that could not be predicted from their independent characterization with, respectively, a reference Rx and a reference Tx. Importantly, the 2.65 dB Rx penalty experimentally determined in section III for the 25 Gbps data rate fully accounts for data rate dependent penalties not seen in the optical Q-factors. It is not cumulative with the data rate dependent penalty observed in section II.B during Tx characterization. This is consistent with the hypothesis of the latter being related to system jitter or to the test environment: As the Rx chip contains a CDR and its electrical output is reshaped, it is much less sensitive to PPG/Tx and ED jitter (the former being compensated by the Rx CDR and the latter having much less impact on the sampling of a reshaped data stream with flat-top symbol shapes).

**Figure 14:**
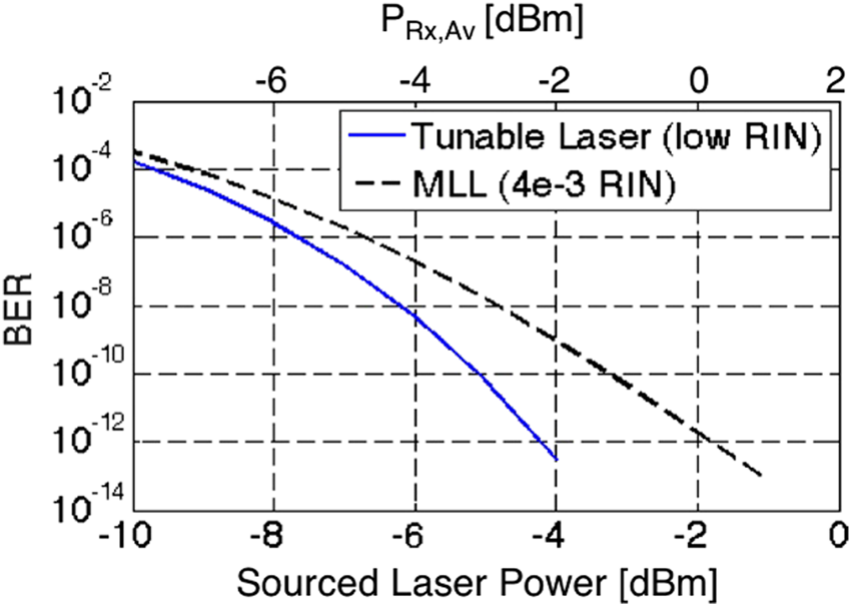
Modelled BER as a function of sourced laser power (lower x-axis) and the average power at the Rx (upper x-axis, after 24 dB amplification by the SOA) for the setup shown in Fig. 12 at a data rate of 14 Gbps, both assuming a low RIN tunable laser (same model as in Fig. 13) and an MLL comb line with an integrated RIN of 4 × 10^−3^.

Since at the optical power levels at which error free operation is reached the average power arriving at the Rx after amplification (2 dB above the sourced laser power indicated on the lower x-axis of Fig. 13(b)) is significantly higher than the Rx sensitivity floor (Fig. 10) and since the utilized tunable laser has very low RIN, the link performance is completely determined by ASE noise at 14 Gbps and by ASE noise and the 2.65 dB Rx penalty at 25 Gbps. Had we been able to do this measurement with the semiconductor MLL instead of the tunable laser, i.e., had the RRM been tunable on the evaluation board, it is obvious that error free operation would not have been reached at 25 Gbps without FEC, since the required comb line power is already at the upper end of the MML’s capabilities even prior to taking RIN into account. However, an uncorrected BER below 10^−12^ appears to be achievable at 14 Gbps with typical comb line characteristics (0 dBm comb line power and an integrated RIN of 4 · 10^−3^), as predicted by the link model once RIN is added back in: Fig. 14 shows the modeled BER as a function of sourced laser power, both with and without the typical comb line RIN of the MLL factored in. Even after factoring in the 1.4 dB insertion losses of the wideband filter, assumed to be reinserted into the link, it is apparent that an MLL comb line power of −0.4 dBm would be sufficient to sustain error free operation at 14 Gbps. Error free back-to-back operation at 25 Gbps in the current configuration with this MLL would require FEC with a coding gain of 5 dBQ.

While there is currently very little link margin in regards to required laser power (0.4 dB factoring in the losses of the wideband filter), since a typical comb line power is 0 dBm, one should bear in mind that the power arriving at the Rx is significantly above its sensitivity floor, due to the high gain of the SOA, so that the noise budget is presently dominated by ASE and RIN. Substantial signal attenuation can be tolerated before receiver noise plays a substantial role. At 0 dBm comb line power, the BER would be expected to be 3 × 10^−13^ back-to-back factoring in RIN and the 1.4 dB insertion losses of the wideband filter. Further 6 dB fiber link attenuation could be tolerated before Rx noise reduces the BER to 10^−12^. On the other hand, at 0 dBm comb line power, there is virtually no margin for dispersion induced vertical eye closure. While measurements indicate modulation with RRMs creates no significant eye closure up to 10 km transmission when operated at the low 2 V_pp_ drive voltage implemented here (see Supplementary Materials), this could rapidly turn into an issue. One should bear in mind though that the target here is a raw BER of 10^−12^ without any FEC. The required link margin to allow for dispersion induced vertical eye closure when servicing longer distances could be provided by FEC coding gain, particularly since the incurred additional latency would then be of lower importance.

Finally, in order to verify compatibility with longer pattern lengths and 64b/66b encoding, we retook the data with PRBS 2^31^–1 data streams (see IEEE standard 802.3 clause 83.5.10), with results also shown in Fig. 13(b). No significant differences were observed in the measured BER. This insensitivity to pattern lengths is not surprising as the selected electronic chipsets are 100 G Ethernet compatible and the optical part of the link was operated in the linear regime (no SOA saturation effects).

## Conclusions

In conclusion, we have experimentally investigated an optical link in single channel configuration consisting of a single section semiconductor mode-locked laser, a resonant ring modulator, an SOA and germanium photodetectors or flip-chip photodetectors. 14 Gbps is reliably supported error free (BER below 10^−12^) for the 8 consecutively tested MLL comb lines. While 25 Gbps appears more challenging, the current performance gap is not very large, so that it could be practical with some improvements or a modest amount of FEC. The experiments reported here still have some shortcomings, such as lack of polarization diversity management (the links were implemented with polarization maintaining fiber), the implementation of optical filters as external, fiber coupled devices, and, most importantly, the restriction to single channel operation. They serve, however, as a good basis for a modelling based evaluation of a full WDM link that will guide future MLL based transceiver development.

## Electronic supplementary material

Supplementary Materials
