# Supplementary material for: Silicon Photonics Transmitter with SOA and Semiconductor Mode-Locked Laser
Source: Sci Rep. 2017 Oct 24;7:13857. doi: 10.1038/s41598-017-14347-3 (PMC5654767; doi:10.1038/s41598-017-14347-3)
Supplement: Supplementary file 1 — Supplementary Materials [file 41598_2017_14347_MOESM1_ESM.pdf]

Silicon Photonics Transceiver with SOA and Semiconductor Mode-Locked Laser

Supplementary Materials

Alvaro Moscoso-Mártir[1], Juliana Müller[1], Johannes Hauck[1], Nicolas Chimot[2], Rony Setter[3], Avner Badihi[3], Daniel E. Rasmussen[3], Alexandre Garreau[2], Mads Nielsen[3], Elmira Islamova[1], Sebastián Romero-García[1], Bin Shen[1], Anna Sandomirsky[3], Sylvie Rockman[3], Chao Li[4], Saeed Sharif Azadeh[1], Guo-Qiang Lo[4], Elad Mentovich[3], Florian Merget[1], François Lelarge[2], and Jeremy Witzens[1]

[1]Institute of Integrated Photonics (IPH) of RWTH Aachen University, Sommerfeldstr. 24, D-52074 Aachen, Germany. E. Islamova is now with Innolume GmbH.
[2]III-V Lab, Campus de Polytechnique, 1 av. Augustin Fresnel, F-91767 Palaiseau Cedex, France. N. Chimot is now with 3SPTechnologies. F. Lelarge is now with Almae Technologies.
[3]Mellanox Technologies, Hakidma 26, Ofer Industrial Park, Yokneam, Israel.
[4]Singapore Institute of Microelectronics (IME)/A*STAR, Science Park Road 11, Singapore Science Park II, Singapore 117685.

These supplementary materials contain two sections: A first section on the optical and Electro-Optical (E/O) devices used in the system experiments (mode-locked laser, resonant ring modulators, flip-chip photodetector subassemblies and integrated germanium photodetectors), as well as a second section with additional data supporting a discussion in regards to the discrepancies between Q-factor and Bit Error Ratio (BER) measurements during Transmitter (Tx) characterization.

I. Devices

1) Semiconductor Single Section Mode-Locked Laser

As already mentioned in the main paper, choosing a passively mode-locked, single section Mode Locked Laser (MLL) as a WDM light source has the advantage of providing a compact and power efficient solution with a fixed carrier grid, but also presents additional challenges in regards to Relative Intensity Noise (RIN) and operational stability. It is a well-known fact that the individual lines of a Fabry-Perot laser cannot serve as independent optical carriers due to mode partition noise resulting in excessive RIN: While the total power emitted by a Fabry-Perot is relatively stable (low RIN as measured over the entire spectrum), the optical power contained in individual lines undergoes strong fluctuations as the total power is dynamically reallocated between them. Mode partition noise is largely suppressed in MLLs by means of the mode locking. Recently, we have been able to measure RIN as low as -120 dBc/Hz decaying to its shot noise limit above 4 GHz on isolated comb lines of a single section MLL provided by III-V Lab [49]. A further characteristic of mode locking in single section MLLs is a flattening of the laser spectrum [SM1] which facilitates providing carriers for a substantial number of WDM channels with a well-defined optical power. Unfortunately, while the spectrum flattens overall over the entire center region of the comb as it changes from a bell shaped to a flattop distribution when entering an injection current / temperature range in which the laser mode-locks, the power variations between adjacent channels also become more pronounced and can exceed 1 dB. An example where this effect is relatively pronounced is shown in Fig. SM1. It is analogous to what is also observed in δ-Δ matched combs generated by parametric processes in microresonators [SM2].

With the current state of the art of semiconductor MLLs, in order to obtain optimum locking performance, the injection current and temperature set points of the laser have to be predetermined (Fig. SM2) prior to performing system level experiments (and prior to operating a transceiver module). An outstanding challenge for future MLL designs is to reliably and repeatedly obtain mode locking in *predetermined* current and temperature ranges, a breakthrough that would also greatly facilitate taking such a technology to production. Integration of Distributed Bragg Reflectors (DBR) on chip [SM3], [SM4] provides additional degrees of design freedom such as tailoring the dispersion and width of the reflection frequency band [SM5] and may be conducive to reaching this objective, as it will greatly reduce the sensitivity of laser characteristics on cleaving accuracy.

The Radio Frequency (RF) linewidth of the laser results from the beat note between adjacent comb lines and is a measure of the degree of correlation of their phase noise. A narrow RF linewidth is the primary indicator of mode locking and correlates with reduced single line RIN. Figure SM2 shows the correlation between the RF linewidth and RIN for the laser reported in [49] (here and in the rest of the paper the integrated RIN is reported as $RIN_t = \sigma_P^2/P_{AV}^2$ where σ_P is the standard deviation (std. dev.) of the optical power and P_{AV} is the average optical power). The first

graph (a) compares the RF beat note spectrum (color plot) with the integrated RIN of the entire comb (blue curve) for different injection currents. When the RF beat-note is well defined and the corresponding linewidth small (visible as a clear yellow line in the color plot), the comb RIN is also low. On the other hand, regions with a smeared out beat note (poor or no mode locking), transitions between mode locking regimes (suddenly changing Free Spectral Range (FSR) for small current or temperature changes), or coexistence of two RF lines [SM6] result in a high RIN. The next graph (b) shows both the comb RIN (RIN taken over the entire optical spectrum), as well as the RIN of an isolated comb line in a zoomed in injection current region. A correlation between all three characteristics – RF linewidth, comb RIN and single line RIN – is clearly visible.

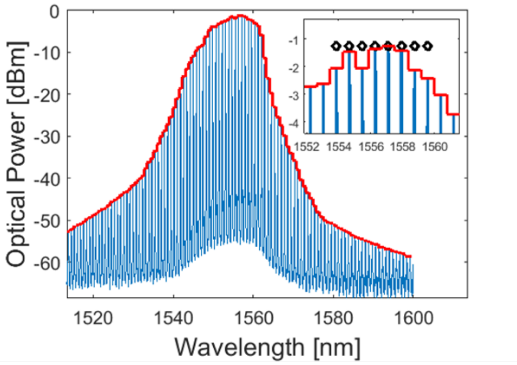

(a)

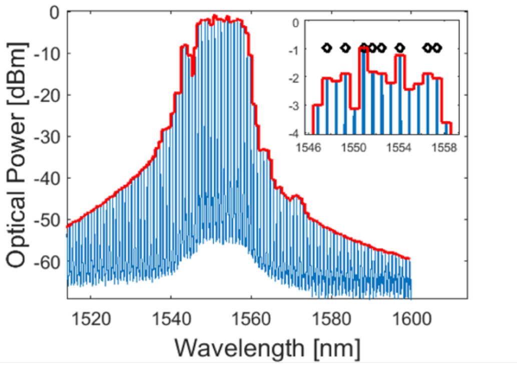

(b)

Fig. SM1. Comparison between the optical spectrum of a single section MLL in a temperature and current range in which it is not mode locking, 300 mA and 30 °C (a), and a current and temperature range in which it is locking, 250 mA and 30 °C (b). The black markers in the insets represent the eight carriers with the highest line power. The laser diode gain material and laser stripe geometry are identical to the MLL reported in [49] but the length of the chip adjusted to obtain a 100 GHz FSR. The 51 pm Resolution Bandwidth (RBW) of the measurements was sufficiently wide for the peak power levels to correspond to the total power of the comb lines.

The characteristics of the 100 GHz FSR laser chosen for the system experiments reported here [SM7] are shown in Fig. SM3. It combines both relatively high power per line (on the order of 0 dBm after coupling to a lensed fiber) and moderate RIN (if somewhat higher than for the laser reported in [49]). The MLL is a Quantum Dash (Q-Dash) single section MLL developed and fabricated at III-V Lab. It is based on a Buried Ridge Stripe (BRS) Fabry Perot cavity with a ridge width of 1.25 µm whose gain material consists in six layers of InAs Q-Dashes in an InGaAsP barrier grown on an InP wafer. The rear facet of the laser is provided with a highly reflective thin film coating, while its front facet is as cleaved. After characterizing the laser at different temperatures and injection currents a good operating point was identified at 25°C and 238 mA, that was then used for subsequent system measurements. At this operating point the laser has a FSR of 102.6 GHz and a center wavelength of 1542 nm. Fifteen consecutive lines have power levels between -1.3 and +1.7 dBm and the total power of the comb is 11 dBm, both measured after coupling to a lensed fiber followed by an isolator. A picture of (a) the laser coupling setup, (b) the laser spectrum and (c) the RIN spectrum for an isolated central line can be seen in Fig. SM3. A more systematic study of the RIN per line is reported in section II.A (Fig. 2) in the main text of the paper.

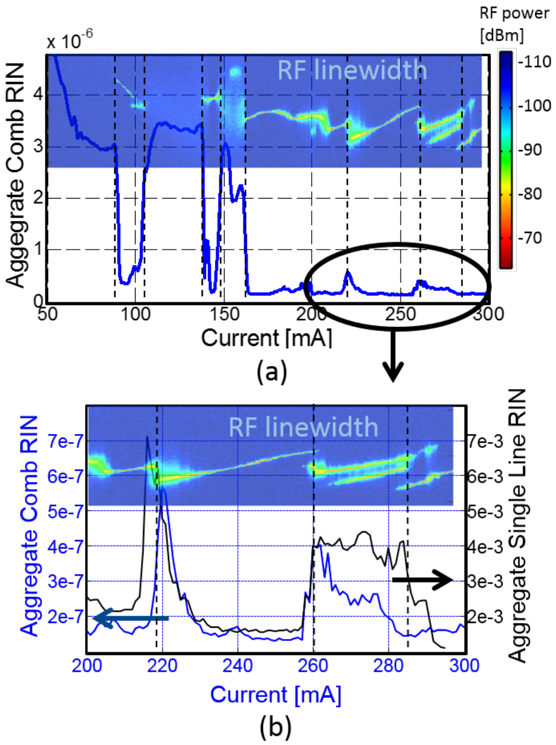

Fig. SM2. (a) RIN of entire comb (blue curve) and spectrum of the RF beat note (color plot). The spectrum of the RF beat note is shown in the frequency range from 60.8 to 60.85 GHz (unlabeled y-axis of the color plot) for the MLL from [49]. The aggregate comb RIN (blue curve) was integrated from 8 to 300 MHz. (b) RIN of the entire comb (blue curve), RIN of an isolated comb line (black curve) and spectrum of the RF beat note (color plot, same frequency range as in (a)) in a zoomed in injection current range. The aggregate comb RIN was integrated from 8 to 300 MHz as in (a) and the single line RIN was integrated from 5 MHz to 20 GHz and represents the total RIN of the line since the RIN spectrum rolls off and reaches shot noise levels above 4 GHz. A clear correlation between mode locking (as evidenced by a single narrowband RF line), reduced comb RIN and reduced single line RIN is apparent, confirming mode locking to be a prerequisite for the usability of isolated comb lines as optical carriers. This data was taken at a laser operation temperature of 30°C.

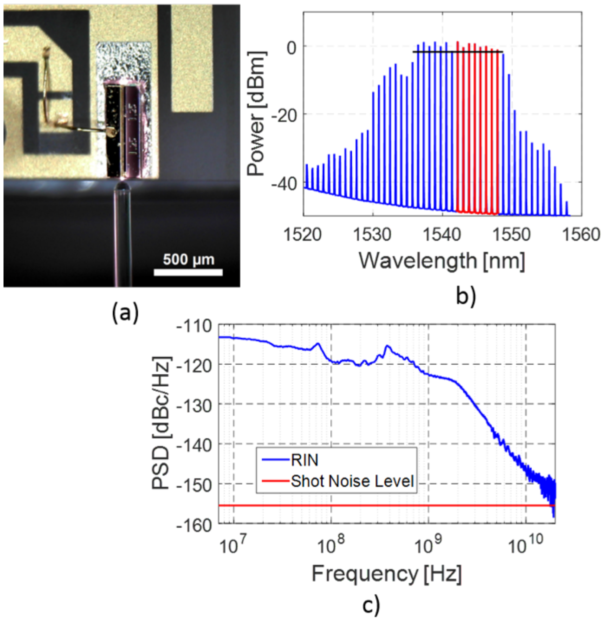

Fig. SM3. MLL mounted on a ceramic submount and coupled to a lensed fiber (a), its recorded optical spectrum (b) and the RIN spectrum of one of the central lines (c). In (b) the black line marks the 15 lines within 3 dB of the peak line power. The 8 lines for which signal Q-factors were measured in the system experiments reported below are shown in red. In (c) the RIN power spectral density of the line with center wavelength at 1546 nm is shown. The RBW of the optical spectrum measurement in (b) was sufficiently wide for the peak power levels to correspond to the total power of the comb lines.

2) *Resonant Ring Modulators*

The Resonant Ring Modulators (RRMs) used for the system experiments have a radius of 10 µm, resulting in a FSR of 10 nm. In addition to the main bus waveguide, a drop waveguide with a low coupling coefficient serves as a tap that can be used to monitor the operating point of the modulator. Modulation is achieved based on the plasma dispersion effect via a phase shifter implemented as a reverse biased pin diode (with a series resistance of 53 Ω and a capacitance of 39 and 29 fF at respectively 0 and 2 V reverse bias). More detailed information on the modulator design, which was specifically targeted for this system architecture, can be found in [49] (in which it is referred to as the "third category of devices").

Two Tx chips are used for the system experiments, which turned out to have somewhat different characteristics due to fabrication variability: Modulators on the first chip were found to have a loaded resonator Quality Factor (Q-factor) of Q_{load} = 5050 (RRM1), while RRMs on the second chip have a reduced Q_{load} of 4300 due to a slightly higher waveguide coupling coefficient as well as higher waveguide losses (RRM2). We define their Modulation Penalty (MP) as $-10log_{10}\left(\frac{P_1-P_0}{P_{in}}\right)$, with P_1 and P_0 the power of the 1- and 0-bit-states inside the bus waveguide right after the RRM and P_{in} the power inside the bus waveguide right before the RRM. It corresponds to the reduction of the Optical Modulation Amplitude (OMA) due to the finite voltage (2 V_{pp}) available to drive the RRM. At the laser frequency to RRM resonance frequency detuning (the "optical carrier detuning") optimized to result in the highest OMA and for a 2 V_{pp} drive voltage the MP is 6.4 dB and 7 dB, respectively for the first and second chip. On the other hand, the combined Grating Coupler (GC) and on-chip bus waveguide losses, respectively 10.3 dB and 9.7 dB for the two chips, are 0.6 dB better for the second chip, resulting in similar output OMAs. This information is provided here since it is relevant for the comparison of individual test results reported in the main parts of the paper – the first chip was used for the Tx characterization with MLL and instrument grade bench top driver electronics (section II.A) while the second chip was wire bonded to chip-scale Tx electronics and characterized with a bench top tunable laser (section II.B). It should also be noted that GCs were not fully optimized in this chip iteration and we have improved them since from ~5 dB IL per GC to better than 3.5 dB in a later chip iteration fabricated in the same full process flow. These improved Insertion Losses (ILs) are further reduced to 3 dB after permanent attachment of a fiber array with index matched epoxy, i.e., 4 dB of link budget improvement would be attainable with this improvement alone. This improved GC design was also used for the Receiver (Rx) chips described in the next subsection and in section III of the main text.

In the case of an unamplified system in which noise is typically dominated by additive Rx noise, the optimum operating point of a modulator corresponds to the highest achievable OMA (assuming the resulting modulator cutoff frequency to also be sufficient, since the latter also depends on the optical carrier detuning in the case of an RRM). On the other hand, in an amplified system signal extinction also matters and the best operating point is between the points with the highest OMA and the highest extinction. Indeed, the signal-ASE beat noise resulting from ASE generated by an optical amplifier also depends on the signal level, so that full extinction is desirable to reduce the zero-level noise, resulting in an additional RRM performance metric in addition to MP and bandwidth. Moreover, higher extinction also reduces the effect of 0-level RIN and allows increasing the channel count while maintaining optical power levels below or at the onset of Semiconductor Optical Amplifier (SOA) saturation. Higher extinction (defined as $10log_{10}\left(\frac{P_1}{P_0}\right)$) is achieved by reducing the optical carrier detuning at the cost of an increased MP and a reduced E/O bandwidth [28] (reported as the -3 dBe cutoff frequency, i.e., the frequency at which the E/O S_{21} has dropped by 3 dBe). This is shown in Figs. SM4 and SM5, in which extinction, MP and bandwidth are plotted versus detuning. While the MP and extinction of RRM1 (Fig. SM4(a)) were measured under DC-conditions, this was not possible for RRM2 (Fig. SM4(b)) since it is wire bonded to an AC coupled modulator driver cutting off the signal at low frequencies (below 100 kHz). Thus, its characteristics were obtained by analyzing eye diagrams resulting from modulation with a 4 Gbps Pseudorandom Bit Sequence (PRBS), which is quasi-DC given the high bandwidth of the Tx subsystem. At higher data rates the modulator cutoff frequency also has to be taken into account to determine the optimum optical carrier detuning. The RRM bandwidth is lowest at resonance ($f_0/2Q$ where f_0 is the carrier frequency and Q is the loaded Q-factor) and increases with detuning due to the emergence of peaking in the E/O S_{21} [28] as can be seen in Fig. SM5. It should also be noted here that since the 0-level ASE noise is particularly sensitive to extinction, getting as close as possible to critically coupled RRMs is much more important here than in an unamplified optical link.

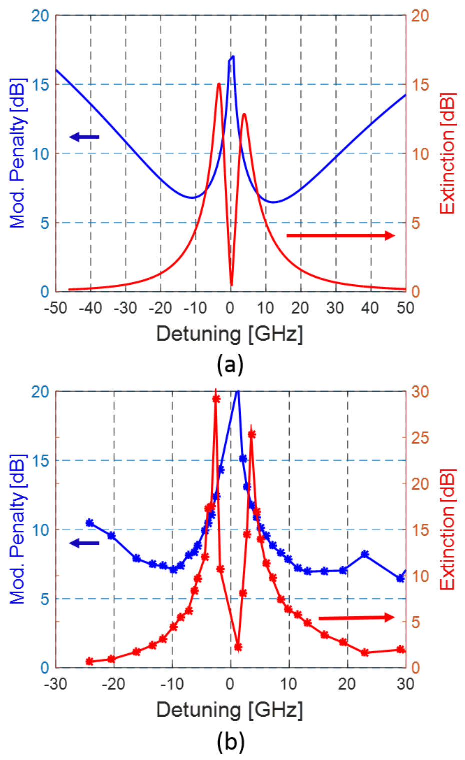

Fig. SM4. RRM extinction and MP vs. optical carrier detuning for chip 1 (a) and chip 2 (b). RRM characteristics are asymmetric in regards to positive and negative detuning due to dynamic free carrier induced absorption losses [28].

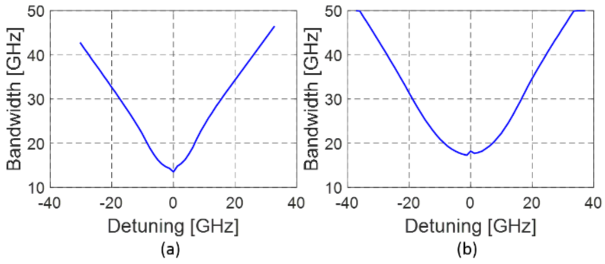

Fig. SM5. RRM bandwidth vs. optical carrier detuning for chips 1 (a) and 2 (b). The cutoff frequency is defined as the point where the E/O S_{21} is 3 dBe below its maximum (more conservative metric than 3 dBe below DC S_{21}).

Between the operating point at which RRM2 has the lowest MP (7 dB at the larger detuning of 18 GHz) and a representative operating point at 7.5 GHz detuning, the estimated RRM cutoff frequency decreases from 32 GHz to ~20 GHz and the measured MP increases from 7 dB to 8.4 dB, but the extinction also increases from 4.8 dB to 8.9 dB. This latter operating point is representative for the system level experiments described in section II.B as it is close to the detuning resulting in optimum optical signal quality at the output of the Tx+SOA, as quantified by the signal Q-factor.

It should also be noted that in all the system experiments reported in this paper we work with positive optical carrier detunings, i.e., the frequency of the laser is above the resonance frequency of the RRM. The reason is twofold: First, this results in a slightly higher OMA, as the contributions to the OMA of dynamic waveguide losses occurring inside the RRM as the free carrier density is modulated and of the refractive index change stack up with the same sign. The other reason is that bistability and self-pulsation occur at negative optical carrier detuning [SM8], thus limiting the maximum optical input power to the RRM in the absence of a control system or a fast offset compensation in the Rx. Even then, dynamic suppression of these instabilities might result in a challenging control problem.

3) *Flip-Chip Photodiodes*

For the realization of the Flip-Chip Photodetector (FC-PD) based Rx, we have opted for the PDCA04-20-SC InGaAs/InP front side illuminated and front side contacted 1x4 photodiode array from Albis Optoelectronics. This

component is flip-chipped onto the Silicon Photonics (SiP) Rx chips and the 20 µm diameter optical aperture of the photodiodes illuminated by means of a GC. It features a typical responsivity of 0.8 A/W in the C-band, a high bandwidth of typically 20 GHz (limited by its RC time constant assuming electrical probing in a 50 Ω environment) sufficient to support 28 Gbps serial data rates and a low capacitance for a vertical incidence photodiode smaller than 100 fF.

The main challenge associated with the integration of this component is the alignment of the light sensitive areas of the photodiodes with the beams emitted by the GCs and minimization of resulting optical losses. Since the beams exit the GCs at a finite angle from normal incidence (16° in air) the optimum FC-PD to GC alignment also depends on the height of the bump bonds used for the flip-chip attachment. To accommodate different flip-chip attachment processes, we fabricated a number of test structures (Fig. SM6) with the position of the GC array offset by different amounts relative to the bump bonding pads. Furthermore, we simulated the RF properties of the assembled Rx and optimized the electrical routing of the photodiode signals to the edge of the SiP chip by tailoring the transmission lines so as to minimize electrical losses, impedance discontinuities and cross-talk (to better than -40 dBe). After a few iterations, we have developed an attachment process yielding reproducible results based on 20 µm high SnAu bump bonds.

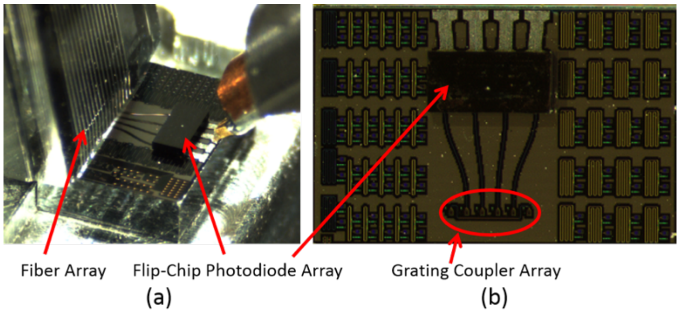

Fig. SM6. (a) Optical probing of the SiP chip with a fiber array. The FC-PD is mounted on the SiP chip and electrically contacted with RF probe tips. (b) Detailed view of the FC-PD array and SiP waveguides routed to a GC array.

A photograph of an assembled SiP chip can be seen in Fig. SM6. We have measured a reliable external (compound) responsivity (normalized relative to the power in the optical fiber) in the range of 0.17 to 0.21 A/W in the large majority of samples. This relatively low responsivity is expected as we are using two GCs (~6 dB compound losses in this chip design) to route the light to the FC-PD. After deembedding GC losses, we obtain a FC-PD responsivity of about 0.84 A/W, as expected. No saturation effects were observed up to the highest measured optical power of 1 mW (as launched into the fiber prior to fiber-to-chip coupling), as expected based on the low Rx input powers seen in this architecture. Besides, the final bandwidth of the subassembly is typically in the range of 15 to 18 GHz when measured in a 50 Ω RF environment at a 2 V bias (see Fig. SM7), which is sufficient to achieve the targeted serial data rate of 25 Gbps per channel. Consequently, the attachment process and the on-chip transmission lines only introduce a modest degradation of the coupling efficiency and bandwidth.

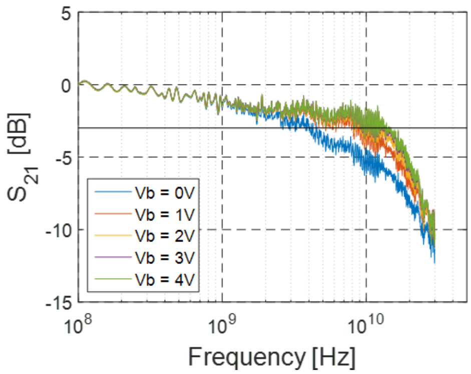

Fig. SM7. E/O S_{21} of the FC-PD.

4) Germanium Waveguide Photodiodes

In parallel, we evaluated SiP Rx chips with monolithically integrated Germanium (Ge) Waveguide Photodetectors (WPDs), with monolithic integration simplifying the assembly as well as reducing optical losses and electrical parasitics [42]. Our design is based on a reverse biased vertical P(Si)-I(Ge)-N(Ge)-junction stack (Fig. SM8). The key requirements for this component are a high bandwidth, a high responsivity and a low dark current. However, these features impose some design constraints that made their mutual optimization challenging in the chosen process line at the Singapore Institute of Microelectronics (IME), A*STAR. The main challenge associated with the design of the Ge WPD was to obtain a sufficiently high cutoff frequency for 25 Gbps serial data rates, due to layout constraints associated with the metal contacts dropped onto the selectively grown Ge pads. These contact pads are required to be at least 2.4 µm to a side, constraining the sizing of the Ge pads and consequently increasing the series resistance associated to electrical transport through the underlying p-doped Silicon (Si). This leads to an increased RC time constant, which turned out to be the limiting factor for the photodiodes' cutoff frequency. Reduction of this series resistance requires higher doping of the Si, which in turns results in deterioration of the material quality of the Ge overgrowth and increases dark currents. Seeking a good tradeoff between these constraints, we moderately increased the p-doping of the underlying Si from the process standard to a peak implanted dopant concentration of 3×10^{19} cm^{-3}.

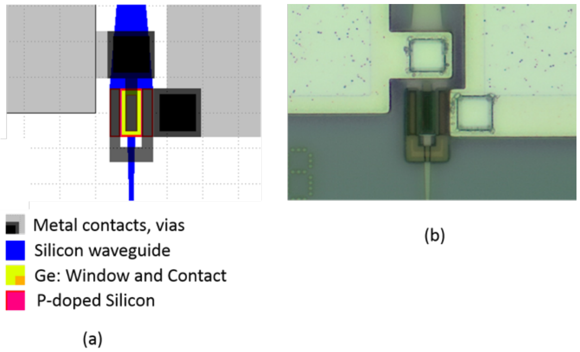

Fig. SM8. (a) Layout and (b) micrograph of the Ge WPD.

The Ge slab is selectively grown on the unetched Si slab and starts with an abrupt optical junction forming a discontinuity in the vertical cross-section of the waveguiding structure. The 800 nm thick Si + Ge slab is also multimode in the vertical direction, supporting a ground mode primarily confined inside the Ge as well as a first order mode with two vertical lobes. Consequently, a beating pattern arises after the abrupt interface, wherein the light periodically moves up and down between the Si and the Ge [42]. This does not only decrease the effective overlap of the light with the Ge and increase the absorption length in the device, but also allows the light to leak out of the Ge slab region when it happens to be primarily located in the Si region at its edges. This might lead to some reduction in responsivity. Side trenches etched into the Si at the edges of the Ge stripe would lead to additional optical confinement. However, they would also further increase the series resistance to the Si contacts, so that we opted not to implement them. Furthermore, the Si-Ge heterointerface is well known to form a barrier for holes transported from the Ge to the Si region, reducing the electrical collection efficiency and increasing carrier recombination at the boundary between the two semiconductors.

Considering these constraints we have designed our vertical junction Ge WPD as follows: The input waveguide is first tapered to a width of 2 µm before merging to a slab of p-doped Si (dose: $5\cdot10^{14}$ cm^{-2} at 20 keV). A stripe of low-temperature Ge with a width of 4.5 µm and a length of 12.4 µm is selectively grown in a window opened in the dielectric films over the slab-region. The top part of the Ge stripe is then n-doped using a shallow Phosphorous implantation process completing the vertical PIN-junction (dose: $4\cdot10^{15}$ cm^{-2} at 10 keV). Highly p-doped wells are defined in the Si for p-side contacting on both sides of the Ge stripe with a spacing of 200 nm from the Ge edge (dose: $4\cdot10^{15}$ cm^{-2} at 20 keV). Finally, the n-doped Ge is contacted by dropping contact plugs directly onto the stripe.

Multiple photodiodes have been measured showing a good performance with a deembedded on-chip WPD responsivity of 0.67 A/W at 1550 nm (extracted from the 0.31 A/W external responsivity shown in Fig. SM9(a)) typical for vertical heterojunction Ge photodiodes [SM9] (even though improved responsivities have also been achieved [SM10], [SM11]). Here too, no saturation effects were observed up to 1 mW fiber power, the maximum

measured optical power in the responsivity curve. The bandwidth is RC-limited and in excess of 30 GHz at 1V reverse bias when measured in a 50 Ω RF environment (see Fig. SM9(b)). At 2 V, the WPD capacitance is measured as 14.2 fF based on breakout structures with larger surfaces (and thus a reliably measurable capacitance). However, a typical dark current of 0.7 μA has been measured at 2 V reverse bias. From these results, it is clear that the bandwidth is much higher than needed for 25 Gbps, so there is some margin to sacrifice bandwidth and improve the other performance metrics for future 25 Gbps chip iterations (in particular reduce again the dark current by reducing the doping of the underlying Si, a critical factor for the material quality of the overgrown Ge).

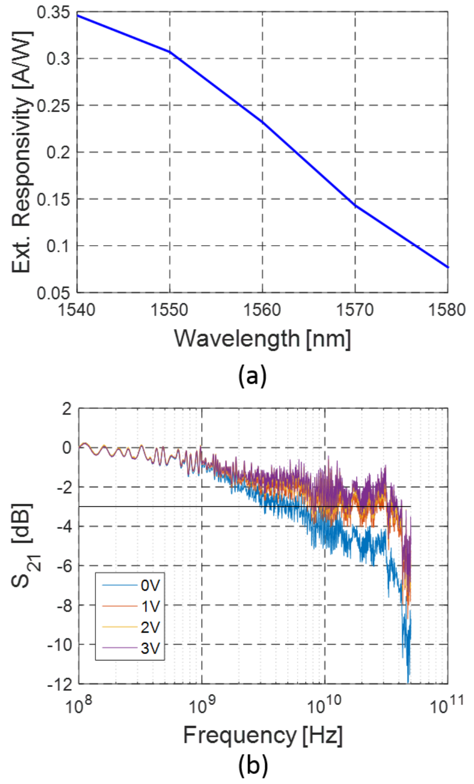

Fig. SM9. (a) External (compound) responsivity (normalized relative to the power in the optical fiber) and (b) E/O S_{21} of the Ge WPD probed in a 50 Ω environment.

Concerned by the high dark current of the Ge WPDs, we characterized their noise spectrum in order to ensure that flicker noise is not an issue. We measured their noise under different illumination conditions as well as with and without voltage bias. For this purpose, we contacted the photodiode chips with RF-probes and biased them to 2 V reverse bias with a bias-T. No flicker noise was discernible above the noise floor of the measurement down to the 40 kHz low frequency cutoff of the bias-T. From the noise floor of the measurement we conclude that the flicker noise Power Spectral Density (PSD) has to be below $4 \cdot 10^{-11}[\mathrm{mW}]/f_s$ (where f_s is the RF signal frequency) and that the std. dev. of the total flicker noise current integrated above 40 kHz (which is below the lower cutoff frequency of the channel, 100 kHz) has to be below 0.1 μA. Given the input referred noise of the Transimpedance Amplifier (TIA) (see section III of the main text), the WPD flicker noise thus does not play a significant role in the Rx sensitivity notwithstanding the high dark current of the devices and the reduced Ge material quality resulting from heteroepitaxy [SM12]. The sensitivity floor of the noise measurement was however not sufficiently low to rule out that other forms of broadband excess noise associated to the material quality, such as increased generation-recombination noise, do not play a role.

II. Discussion of Discrepancies between Q-Factor and BER Measurements

For the measurements reported in section II of the main text, the Bit Error Ratio (BER) was recorded for an optimized sampling time and threshold (matching the assumptions used for the Q-factor extraction). However, as we can see in

Fig. 6(b), there are significant discrepancies at both 14 and 25 Gbps between the measured BER and the BER predicted based on the measured Q_{sig} (corresponding to respectively +1.2 dBQ and -2 dBQ, wherein Q[dBQ] is defined as $20log_{10}(Q_{sig})$). While the BER at 25 Gbps is worse than expected, it is, surprisingly, better at 14 Gbps. We did a series of characterization measurements in order to better understand the possible sources of discrepancies:

For one, we verified whether the simplification consisting in a Gaussian noise model when predicting BER from a recorded Q-factor could be an important factor. While it is a well-known fact that the assumption of Gaussian noise statistics is not accurate for ASE noise and leads in particular to the wrong prediction in regards to the optimum decision threshold [SM13], the discrepancy in regards to the predicted BER should be very slight and not sufficient to explain what we observe here [SM13], [SM14]. In order to verify this, we recorded the BER for different decision thresholds for a laser power achieving a BER in the range of 10^{-12} at the optimum threshold (blue curve in Fig. SM10(a)). The recorded data was then fitted assuming non-Gaussian Chi square noise statistics and the extinction recorded from the experiment (red curve). The black curve in Fig. SM10(a) shows the BER as a function of decision threshold assuming Gaussian noise statistics with the same 0- and 1-level ASE noise std. devs. As can be seen, the model based on non-Gaussian statistics is in close agreement with the recorded data, while the optimum threshold predicted by the Gaussian noise model is significantly off. Nevertheless, the resulting BER is nearly the same for both models. The excess noise of the oscilloscope was also independently measured and found to be negligible in these experiments. The 1.2 dBQ discrepancy at 14 Gbps may be a penalty associated with recording the eye diagrams with a 20 GHz real-time oscilloscope with a square transfer function: This slightly increases ISI and worsens ASE-signal beat noise [51].

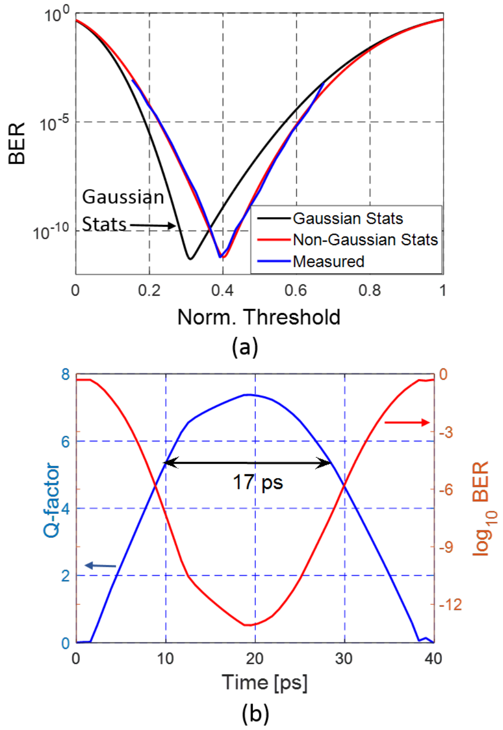

Fig. SM10. (a) Vertical Bathtub Curve: BER measured at 14 Gbps as a function of decision threshold (blue curve), model assuming the non-Gaussian noise statistics from [SM14] (red curve) and model assuming Gaussian noise statistics with the same 0- and 1-level ASE noise std. dev. (black curve). The normalized decision threshold is indicated relative to the 0- and 1-levels, i.e., a threshold of 0 corresponds to the 0-level and a threshold of 1 to the 1-level. The extinction in this experiment was -7 dB. (b) Horizontal Bathtub Curve showing the signal Q-factor as well as the predicted BER, both extracted from a typical Tx eye diagram at 25 Gbps, as a function of sampling time.

On the other hand, the unexpected -2 dBQ degradation of the BER vs. signal Q-factor seen at 25 Gbps does not seem to be a characteristic of the investigated link, but rather related to the test environment, as it was also seen with the same magnitude in a reference experiment. The latter consisted in cascading a noisy light source (low power laser amplified by an EDFA) with a commercial Mach-Zehnder Modulator (MZM) directly driven by the Programmable Pattern Generator (PPG) and directly fed into the U2T/Finisar Rx without post-modulation optical amplification. This experiment was compared to the investigated link (RRM + Driver Chip + SOA). The laser power was independently

set in both experiments so as to obtain a low speed BER of 10^{-9}. In both cases the BER worsened from 10^{-9} to ~10^{-6} as the data rate was increased from 14 to 25 Gbps.

A possible explanation for the degradation of the BER at 25 Gbps is slowly varying jitter not seen in the oscilloscope traces that are comparatively short relative to the gating periods of the BER measurements (that were done without Clock Data Recovery (CDR) at the Error Detector (ED)). In order to evaluate how much jitter would be needed to explain the difference between the measured Q-factor (Q_{sig}) and the lower Q_{sig} expected based on the BER measurements, we extracted Q-factors from the eye diagrams that were effectively averaged over sampling time ranges according to an assumed peak-to-peak jitter: For each of the sampling times falling within this time range the corresponding BER was first calculated, averaged over the entire range, and then reverse transformed into an averaged effective Q_{sig}. The red dashed line in Fig. 6(b) shows the result when we assume a peak-to-peak time window of 17.2 ps. This amount of jitter is not unlikely since the phase margin of the Bit Error Rate Tester (BERT) is specified as 28 ps at 25 Gbps corresponding to a jitter of 12 ps (the jitter of the PPG alone already accounts for up to 8 ps according to specifications). In the case of the RRM + Driver Chip system experiment, this is further compounded by any jitter generated in the driver chip as well as in the commercial photoreceiver (the latter also applying to the reference measurement). Some additional jitter may also arise from the time delay introduced by the RRM. Since it depends on the optical carrier detuning, it may vary if the optical carrier detuning drifts over time (due to the inaccessibility of the thermal tuner pins, an active control loop of the RRM resonance was not implemented). An exact expression of the time delay can be obtained by taking the derivative of the phase of the RRM's E/O S_{21} [28] in respect to the angular RF modulation frequency and is below 7 ps (the latter corresponding to the time delay at low signal frequencies and zero optical carrier detuning and is calculated as $2Q/\omega_0$, where Q is the Q-factor of the resonator and $\omega_0 = 2\pi f_0$ is the angular carrier frequency). While the RRM cannot significantly drift from its operating point without severely compromising MP and other RRM performance metrics, a slow drift of the time delay by 1 or a few ps is possible, further shifting the BERT sampling time from its optimum in the absence of a Rx CDR. Both experiments, the system experiment with RRM and driver chip and the reference experiment with the commercial MZM, have in common that the BER is quite sensitive on sampling time even close to its optimum and is thus sensitive to timing jitter. An example of a horizontal bathtub curve is shown for the RRM + Driver + SOA system experiment in Fig. SM10(b).

III. Transmission Experiments At Longer Distances

Even though short distance links are the main target of this work, we made transmission experiments at different drive voltage and distances to investigate chirp induced Inter-Symbol Interference (ISI). It is a well-known fact that RRMs modulate both the phase and amplitude of the carrier. Thus, they induce chirp when amplitude modulating. As the drive voltage is increased, the maximum phase change is also increased and chirp worsens.

The experiments were done with RRMs similar to those used for the system experiments reported in the main part of the paper (same design and same fabrication run, different chip). Light was provided by the Keysight tunable laser and the RRMs were driven by the PPG of the BERT after amplification with a high-speed amplifier from Finisar/U2T (Part Number TGA4943-MOD, > 30 GHz cutoff frequency). The wavelength of the tunable laser was chosen as the wavelength resulting in the highest OMA. The gain of the electrical amplifier was chosen to result in the targeted drive voltage swing. With a 2 V_{pp} signal, no additional eye closure was observed for the investigated transmission distances, up to 10 km. For 6 V_{pp}, a slight increase in jitter and a slight vertical eye closure penalty of 0.5 dB was observed. Figure SM11(a) shows the vertical eye opening at 6 V_{pp} drive voltage as a function of fiber length expressed as a fraction of the DC 1- and 0-levels. The normalization to DC signal levels removes the effect of fiber losses (that is not of significance here as it is also partially the result of connector losses at the interfaces between several patch cords), but maintains the effect of dispersion induced inter-symbol interference. Figures SM11(b) and (c) show examples of eye diagrams (as recorded by the linear U2T/Finisar receiver) after 0 and 10 km fiber transmission lengths.

Another mechanism enhancing dispersion induced penalties might consist in the conversion of RIN into phase noise via the linewidth-enhancement factor of the saturating SOA [SM15] when used in multi-channel configuration (in the single channel experiments done in this paper the SOA is not saturating). While a conclusive quantification of this effect at increased fiber lengths will require further work, we expect this effect to be strongly suppressed by the anti-correlation of RIN between multiple comb lines: When measuring the RIN of a large number of comb lines taken together, the resulting RIN drops by several orders of magnitude compared to isolated comb lines [SM16]. Cross-gain modulation (XGM) resulting from the data modulation in a partially saturating SOA, in conjunction to the linewidth-enhancement factor, might however result in increased chirp and higher dispersion penalties.

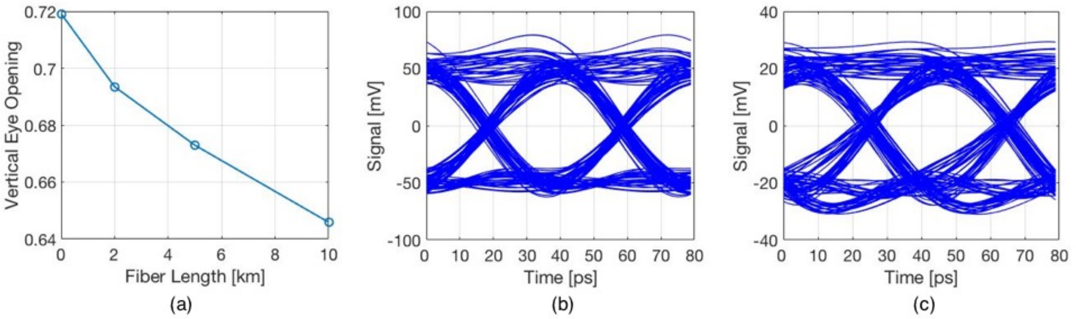

Fig. SM11. (a) Vertical eye opening, as a fraction of the DC 1- and 0-levels, as a function of fiber length for an RRM drive signal of 6 V_{pp}. (b) and (c) show transmitter eye diagrams, respectively after 0 and 10 km fiber lengths, as generated by an RRM driven with a 6 V_{pp} signal. Eye diagrams are averaged over repeating PRBS cycles so as to remove noise but maintain the effect of inter-symbol interference. The commercial photoreceiver used to record the eye diagrams was inverting, so that the optical levels have been flipped.

References:

[SM1] A. Akrout *et al.*, Separate Error-Free Transmission of Eight Channels at 10 Gb/s using comb generation in a quantum-dash-based mode-locked laser. *Photon. Technol. Lett*. **21**(23)*,* 1746-1748 (2009).

[SM2] T. Herr et *al.*, Universal formation dynamics and noise of Kerr-frequency combs in microresonators. *Nat. Photon.* **6**(7), 480-487 (2012).

[SM3] S. Joshi *et al.*, Mode Locked InAs/InP Quantum dash based DBR Laser monolithically integrated with a semiconductor optical amplifier. in *Proc. 25th Int. Conf. on Indium Phosphide and Related Materials (IPRM2013)*, WeD2-4 (2013).

[SM4] S. Joshi *et al.*, Quantum dash based single section mode locked lasers for photonic integrated circuits. *Opt. Express* **22**(9), 11254-11266 (2014).

[SM5] M. J. Strain, P. M. Stolarz, and M. Sorel, Passively Mode-Locked Lasers with Integrated Chirped Bragg Grating Reflectors. *J. Quant. Electron.* **47**(4), 492-499 (2011).

[SM6] K. Merghem *et al.*, Stability of Optical Frequency Comb Generated With InAs/InP Quantum-Dash-Based Passive Mode-Locked Lasers. *J. Sel. Top. Quant. Electron.* **50**(4), 275-280 (2014).

[SM7] J. Müller *et al.*, High Speed WDM Interconnect Using Silicon Photonics Ring Modulators. in *Proc. 2015 Europ. Conf. Opt. Comm. (ECOC)*.

[SM8] T. Van Vaerenbergh, M. Fiers, J. Dambre, and P. Bienstman, Simplified description of self-pulsation and excitability by thermal and free-carrier effects in semiconductor microcavities. *Phys. Rev. A* **86**, 063808 (2012).

[SM9] T. Baehr-Jones *et al.*, A 25 Gb/s Silicon Photonics Platform. *arXiv:1203.0767* (2012), http://arxiv.org/abs/1203.0767.

[SM10] Y. Zhang *et al.*, A high-responsivity photodetector absent metal-germanium direct contact. *Opt. Express* **22**(9), 11367-11375 (2014).

[SM11] N.-N. Feng *et al.*, Vertical p-i-n germanium photodetector with high external responsivity integrated with large core Si waveguides. *Opt. Express* **18**(1), 96-101 (2010).

[SM12] L. Colace, A. Scacchi, and G. Assanto, Noise Characterization of Ge/Si Photodetectors. in *Proc. 2011 IEEE Int. Conf. Group IV Photon. (GFP),* 290-292.

[SM13] P. A. Humblet, and M. Azizoglu, On the Bit Error Rate of Lightwave Systems with Optical Amplifiers. *J. Lightwave Technol*. **9**(11), 1576-1582 (1991).

[SM14] N. S. Bergano, F. W. Kerfoot, and C. R. Davidson, Margin Measurements in Optical Amplifier Systems. *Photon. Technol. Lett.* **5**(3), 304-306 (1993).

[SM15] A. D. McCoy P. Horak, B. C. Thomsen, M. Ibsen, D. J. Richardson, Noise Suppression of Incoherent Light Using a Gain-Saturated SOA: Implications for Spectrum-Sliced WDM Systems. *J. Lightwave Technol.* **23**(8), 2399-2409 (2005).

[SM16] J. Müller *et al.* Silicon photonics WDM transmitter with single section semiconductor mode-locked laser. *Adv. Opt. Technol*. (De Gruyter) **4**(2)*,* 119-145 (2015).
